# What is working memory capacity, and how can we measure it?

**DOI:** 10.3389/fpsyg.2013.00433

**Published:** 2013-07-24

**Authors:** Oliver Wilhelm, Andrea Hildebrandt, Klaus Oberauer

**Affiliations:** ^1^Department of Psychology, University of UlmUlm, Germany; ^2^Department of Psychology, Humboldt-Universität zu BerlinBerlin, Germany; ^3^Department of Psychology, University of ZürichZurich, Switzerland

**Keywords:** working memory capacity, fluid intelligence, secondary memory, cognitive control, binding

## Abstract

A latent variable study examined whether different classes of working-memory tasks measure the same general construct of working-memory capacity (WMC). Data from 270 subjects were used to examine the relationship between Binding, Updating, Recall-N-back, and Complex Span tasks, and the relations of WMC with secondary memory measures, indicators of cognitive control from two response-conflict paradigms (Simon task and Eriksen flanker task), and fluid intelligence. Confirmatory factor analyses support the concept of a general WMC factor. Results from structural-equation modeling show negligible relations of WMC with response-conflict resolution, and very strong relations of WMC with secondary memory and fluid intelligence. The findings support the hypothesis that individual differences in WMC reflect the ability to build, maintain and update arbitrary bindings.

The terms *working memory* and *working memory capacity* are used with different meanings in a broad range of research fields. In this paper we use working memory to refer to a hypothetical cognitive system responsible for providing access to information required for ongoing cognitive processes, and we use working-memory capacity (WMC) to refer to an individual differences construct reflecting the limited capacity of a person's working memory. Our aim is to achieve a better understanding of this construct, its measurement, and its relations to other ability constructs.

Various indicators have been developed to capture individual differences in WMC over the last 30 years. The best known and most frequently used class of tasks for measuring WMC is probably the *complex span* paradigm (Daneman and Carpenter, 1980; Conway et al., 2005). Many studies of individual differences in WMC measure this construct exclusively through one or several variants of complex-span tasks. As a consequence, much recent theorizing about what underlies individual differences in WMC has focused—perhaps too narrowly—on the complex span task class (e.g. Unsworth and Engle, 2007a; Barrouillet et al., 2011; Oberauer et al., 2012).

In this article we argue for a broader perspective on WMC as an individual-differences construct. We investigate the relationship between complex-span performance to other indicators of WMC and related constructs. This research has three interlinked aims We evaluate the validity of several task classes for measuring WMC; we test three theories of the nature of WMC; and we study predictions concerning the relation of WMC with other cognitive constructs. We next review the three theoretical views on how to characterize WMC as an individual-differences construct: The executive-attention view of WMC (e.g., Engle, 2002), the primary-memory/secondary-memory view (Unsworth and Engle, 2007b), and the binding hypothesis (Oberauer et al., 2007; Oberauer, 2009). Figure 1 presents an overview of the constructs to be discussed, examples of indicators by which they can be measured, and their relations as postulated by the three theories.

**Figure 1:**
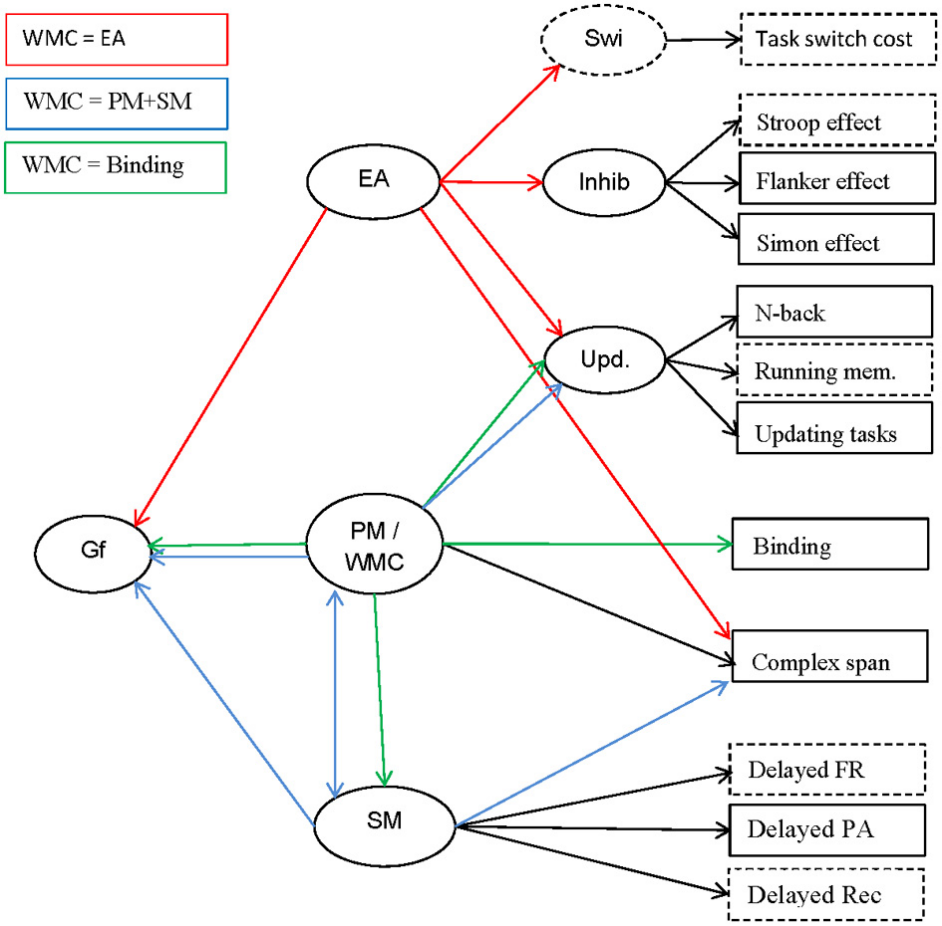
**Schematic outline of the constructs investigated (ovals), their indicators (rectangles), and their relations (arrows).** The relations are color-coded (see legend in upper left corner) to indicate which relation is postulated by which of the three theories of WMC discussed in the text (relations drawn in black are theoretically non-controversial). Relations not coded in the color of a theory might be compatible with the theory in question but are not explicitly assumed in that theory. Indicators and constructs not measured in the present study are drawn with broken lines. Gf, fluid intelligence; EA, executive attention; PM, primary memory; WMC, working-memory capacity; SM, secondary memory; Swi, task switching/shifting; Inhib, inhibition; Upd, working-memory updating; FR, free recall; PA, paired-associates recall; Rec, recognition.

## WMC as executive attention

Engle (2002) hypothesized that WMC “is about using attention to maintain or suppress information” (p. 20). Elsewhere Engle et al. (1999, p. 104) argued that WMC “is not really about storage or memory *per se*, but about the capacity for controlled, sustained attention in the face of interference or distraction.” The executive-attention hypothesis was empirically tested by comparing high- and low-span participants on a variety of paradigms measuring cognitive control. For instance, in one study participants selected for having very high or very low complex-span scores performed a saccade task (Kane et al., 2001). In the prosaccade condition a visual cue was presented on the same side of the screen where the to-be-identified target appeared later. In the antisaccade condition the cue appeared on the opposite side from the target, supposedly distracting attention. Low-span participants were slower and less accurate in the antisaccade condition than high-span participants, but the groups did not differ in the prosaccade condition. Similar results with other executive-control paradigms were found in further studies comparing participant groups with high versus low complex-span scores—for example using the Stroop task (Kane and Engle, 2003), a Dichotic-Listening task (Conway et al., 2001), and a go/no-go task (Redick et al., 2011). These studies are limited in two regards. First, they compare extreme groups of individuals defined by their complex-span scores. Extreme-group comparisons are well suited for detecting individual differences but ill-suited for estimating their effect size, because they tend to overestimate effects in the population. Second, testing executive attention with a single experimental paradigm conflates variance due to individual differences in executive control with task-specific variance.

Both limitations have been overcome in correlational studies that measured WMC and executive attention through multiple indicators and evaluated their relationship through structural equation modeling (SEM). An important foundation for this endeavor has been laid by the seminal SEM study of executive functions by Miyake et al. (2000). They identified three separate, but positively correlated executive-function factors: *inhibition*, (task-set) *shifting*, and working memory *updating*.^1^

More recent studies have related WMC to some of these three executive-function factors. Oberauer et al. (2003) found modest correlations around 0.30 between task-set shifting (referred to as “supervision” in their study) and WMC factors (see also Oberauer et al., 2007). Keye et al. (2009, 2010) found no substantial relation between response inhibition in the Eriksen flanker task and also the Simon task and a latent WMC factor. In contrast, two studies by Unsworth and his colleagues obtained moderate positive correlations between latent factors reflecting executive attention (primarily measured through inhibition indicators) and WMC (Unsworth, 2010; Unsworth and Spillers, 2010). On balance, the evidence points to a positive but not very high correlation between WMC and the shifting and inhibition factors of executive control. One aim of our present study was to contribute further evidence on the correlation between WMC and indicators of inhibition, using latent-factor modeling to isolate different sources of variance in the inhibition indicators.

The relation between WMC and the updating factor of Miyake et al. (2000) is more complex. Whereas shifting and inhibition have been measured by difference scores isolating the executive process of interest by contrasting two experimental conditions, the updating factor of Miyake et al. (2000) has been measured by overall performance in working memory tasks that involve updating. Therefore, performance in those tasks reflects a mixture of WMC and updating ability.^2^ For that reason, working-memory updating tasks have been used not only as indicators of the executive-function factor called *updating* in the Miyake et al. (2000) model (see also Friedman et al., 2008; Miyake and Friedman, 2012), but also as indicators of WMC (Kyllonen and Christal, 1990; Süßet et al., 2002; Oberauer et al., 2008; Lewandowsky et al., 2010). In recent years, a number of studies investigated the relationship between tasks measuring WMC arguably not involving updating, such as complex span tasks, and tasks assumed to strongly involve updating of working memory, such as the *n*-back task. In *n*-back, participants are presented with a long sequence of stimuli and are requested to decide for each stimulus whether it matches the one *n* steps back in the sequence. Several studies reported only small correlations between performance on the *n*-back task and complex span tasks (Kane et al., 2007; Jaeggi et al., 2010a,b). In contrast, Schmiedek et al. (2009) obtained a nearly perfect correlation between a latent factor measured by three complex span tasks and a latent factor represented by three different working-memory updating tasks. The three updating indicators included a figural *n*-back task, the *memory-updating* task (Oberauer et al., 2000) in which participants updated memorized digit values by arithmetic operations performed on them, and a new task called *alpha span* that required continuous updating of the order of to-be-remembered letters. The low correlations between *n*-back performance and WMC measures in previous studies can be attributed to various combinations of three factors: (1) The use of single indicators for measuring updating (i.e., the *n*-back task) and for measuring WMC; (2) the mismatch of content domains between a spatial-figural *n*-back task and verbal complex span tasks (Kane et al., 2007); and (3) the mismatch of the memory-test methods—recognition in *n*-back tasks versus recall in complex span. In the present study we revisit the relationship between complex span and working memory updating tasks, using multiple indicators, balanced across content domains, for both categories of tasks, and consistently testing memory through recall to avoid differences in method variance.

Another question of interest in this context is whether WMC tests with and without updating account for different portions of variance in fluid intelligence. Kane et al. (2007) have found that performance in a complex-span task and in an *n*-back task were largely independent and equally good predictors of fluid intelligence. One reason for this finding could be that the complex-span task used verbal material whereas the *n*-back task used visual-spatial material. Here we use a broad set of updating tasks to test whether they contribute to the prediction of fluid intelligence over and above complex-span tasks.

## WMC as primary and secondary memory

Building on traditional dual-store models, Unsworth and Engle (2007a) proposed that performance in complex-span tasks draws on two sources, a limited capacity component that maintains information over brief periods of time, and a more durable component that stores information over longer time periods. WMC as reflected in complex span performance is thus characterized as a composite of active maintenance (primary memory: PM) and controlled retrieval from secondary memory (SM). SM is critical for performance as soon as the load on PM reaches its capacity limit. PM is argued to have a capacity of about four elements, but in complex span, part of this capacity is required for the distractor task, thereby displacing list items from PM. Therefore, recall in complex span tasks relies to a larger extent on SM than in simple span tasks. Retrieval from SM is a cue-dependent search process that is adversely affected by proactive interference, encoding deficits, and output interference. Limitations in both PM and SM are reflected in complex-span performance. According to Unsworth and Engle, (2007a, p. 1038), “… simple and complex span tasks largely measure the same basic subcomponent processes (e.g., rehearsal, maintenance, updating, controlled search) but differ in the extent to which these processes operate.” Simple-span tasks are supposed to measure predominantly maintenance in PM, whereas complex-span measures capture mainly controlled search in SM.

Based on these assumptions, Unsworth and Engle (2007a) predicted that the correlation between complex span and fluid intelligence is mediated by individual differences in two constructs, the capacity of PM and the efficiency with which individuals encode information into SM and search information in SM. This prediction has been confirmed by several correlational studies (Unsworth et al., 2009; Unsworth, 2010; Unsworth and Spillers, 2010). Other studies provide additional evidence that the acquisition of associations in SM predicts fluid intelligence over and above complex span (Tamez et al., 2008, 2012; Kaufman et al., 2009).

In the present study we therefore included a measure of associative SM to investigate its relation to complex-span performance and to working-memory updating. We predict that SM should be strongly correlated with complex span, in agreement with prior work by Unsworth and his colleagues. We expect that the correlation between SM and working-memory updating should be comparatively smaller because the updating tasks require the maintenance of a small number of items, hardly exceeding the presumed capacity of PM. Moreover, the requirement to rapidly update the remembered items renders SM unsuitable for their maintenance because SM is susceptible to proactive interference, which would build up across multiple updating steps. As a consequence, SM should be virtually useless for updating tasks (see Cowan et al., 2012, for a similar argument). We also asked whether SM contributes to the prediction of fluid intelligence over and above established classes of WMC indicators (e.g., complex span and updating tasks).

## The binding hypothesis of WMC

In our own view, working memory is a system for building, maintaining and rapidly updating arbitrary bindings. For instance, items in a list are bound to list positions, objects are bound to locations in space, and concepts are bound to roles in propositional schemata. The capability for rapid formation of temporary bindings enables the system to construct and maintain new structures, such as random lists, spatial arrays, or mental models. Working memory is important for reasoning because reasoning requires the construction and manipulation of representations of novel structures. The limited capacity of working memory arises from interference between bindings, which effectively limits the complexity of new structural representations, and thereby constrains reasoning ability (Oberauer et al., 2007).

Evidence for the binding hypothesis comes primarily from two sources. First, tasks specifically designed to measure the ability of constructing new structural representations have been shown to be closely correlated with conventional measures of WMC, and to be excellent predictors of fluid intelligence. This is true even for task versions that do not require any memory because all relevant information is constantly visible (Oberauer et al., 2003; Bühner et al., 2005; Oberauer et al., 2008; Chuderski et al., 2012). Second, when short-term recognition performance is decomposed into contributions from familiarity and recollection, the latter, but not the former is correlated with WMC. Recollection, but not familiarity reflects the maintenance of temporary item-context bindings (Oberauer, 2005; Öztekin and McElree, 2010).

According to the binding hypothesis, working-memory updating tasks should be excellent measures of WMC because they involve rapid updating of temporary bindings. Tasks such as the *n*-back task, the *running-memory* task, and the *memory-updating* task (Oberauer et al., 2000) require participants to remember a small number of items in their correct order (in *n*-back and running memory) or in their correct spatial location (in memory updating). Thus, items must be bound to their ordinal positions or their spatial locations. These bindings must be continuously updated. For instance, in the memory-updating paradigm, participants initially encode a set of digits, each in a different spatial location, and then update the values of individual digits by the results of arithmetic operations. Each updating step requires updating of bindings between digits and their locations. According to this view, updating tasks are closely related to other measures of WMC, and to fluid intelligence, not because they reflect executive attention, but because they reflect the maintenance of temporary bindings. Because these bindings must be updated rapidly for multiple times, there is little chance for gradual learning of long-term associations. Therefore, we argue that updating tasks are particularly well suited for measuring people's ability to build and maintain temporary bindings in working memory, with little contribution from associative-learning mechanisms of SM.

## Aims and predictions for the present study

The present study has three interlinked aims. First, we test hypotheses from the three theoretical views about the nature of WMC outlined above. Second, we investigate to what extent different task classes for measuring WMC are interchangeable indicators of the same construct. Third, we explored how WMC is related to SM, cognitive control, and fluid intelligence. The three aims are interlinked because different theories about the nature of WMC lead to different expectations about which kind of tasks measure the same construct (i.e., WMC), and about how these tasks and constructs relate to other cognitive constructs. To this end, we tested participants on multiple tests of the following categories: (1) complex-span tasks (Cspan), (2) working memory updating tasks (Updating), (3) tests of immediate memory for temporary bindings (Binding), (4) tests of SM for associations (SM), (5) tasks measuring response inhibition (Inhibition), and (6) tests of fluid intelligence (Gf).

The executive-attention theory of WMC motivates the following predictions (see Figure 1, red arrows): Cspan tasks should be highly correlated with Updating and with Inhibition, because the latter two represent aspects of executive functions. The common variance of these three classes of measures, reflecting general executive attention, should be a good predictor of Gf. This theory does not rule out that other constructs, such as SM or Binding, also contribute to predicting Gf.

The dual-component theory of Unsworth and colleagues conceptualizes performance in complex-span tasks as being determined by maintenance in PM and search in SM (see Figure 1, blue arrows). As we argued above, tests of working-memory updating are unlikely to rely much on SM, and therefore reflect maintenance in PM to a larger extent than complex-span tasks. Therefore, we can expect that Cspan is related to SM on the one hand, and to PM (as measured by Updating) on the other hand, whereas SM and Updating are comparatively weakly related to each other. Updating and SM should also contribute independently to predicting Gf.

The binding hypothesis implies that a measure of the maintenance of temporary bindings should share a large proportion of variance with other measures of WMC, including Cspan and Updating. The shared variance of all those measures is assumed to reflect the WMC construct (see Figure 1, green arrows). Together with the binding measures, the updating tasks should have comparatively high loadings on this construct because, as we argued above, these tasks require the rapid updating of bindings. The broad WMC construct, reflecting maintenance and updating of bindings, should be a good predictor of Gf. In contrast, Inhibition is not expected to be closely related to Cspan, Updating, or Gf. SM performance should substantially depend upon the ability to create bindings in working memory. According to the binding hypothesis, temporary bindings in working memory, not more long-term associations in SM, are directly relevant for reasoning. Therefore, we expect that Gf is better predicted by WMC (i.e., the shared variance of Binding, Updating, and Complex Span) than by SM.

## Methods

### Participants

The mean age of the final sample (*N* = 262) was 27.41 years (*SD* = 4.83) and 56% were female. The sample had a broadly varying educational background. Thirty-one percent of the participants did not have a high school degree, 47% of the sample had a high school degree but no completed college degree, and 22% of the sample held academic degrees.

### Procedure

Trained research assistants tested up to 9 participants simultaneously. Each participant completed two sessions, both lasting ~3 h including breaks. The time interval between sessions ranged from 4 to 6 days. The order of the tasks was constant across participants.^3^ Each task was instructed directly before administration. Practice trials with feedback about accuracy were completed before administering the tests. There was no feedback for test trials. All computerized tasks were programmed using Inquisit 3.0^©^. Besides the 20 tasks analyzed in the present study participants completed seven gambling tasks, three mental speed tasks and two self-report questionnaires.

### Measures, scores and estimates of reliability

**Figure 2:**
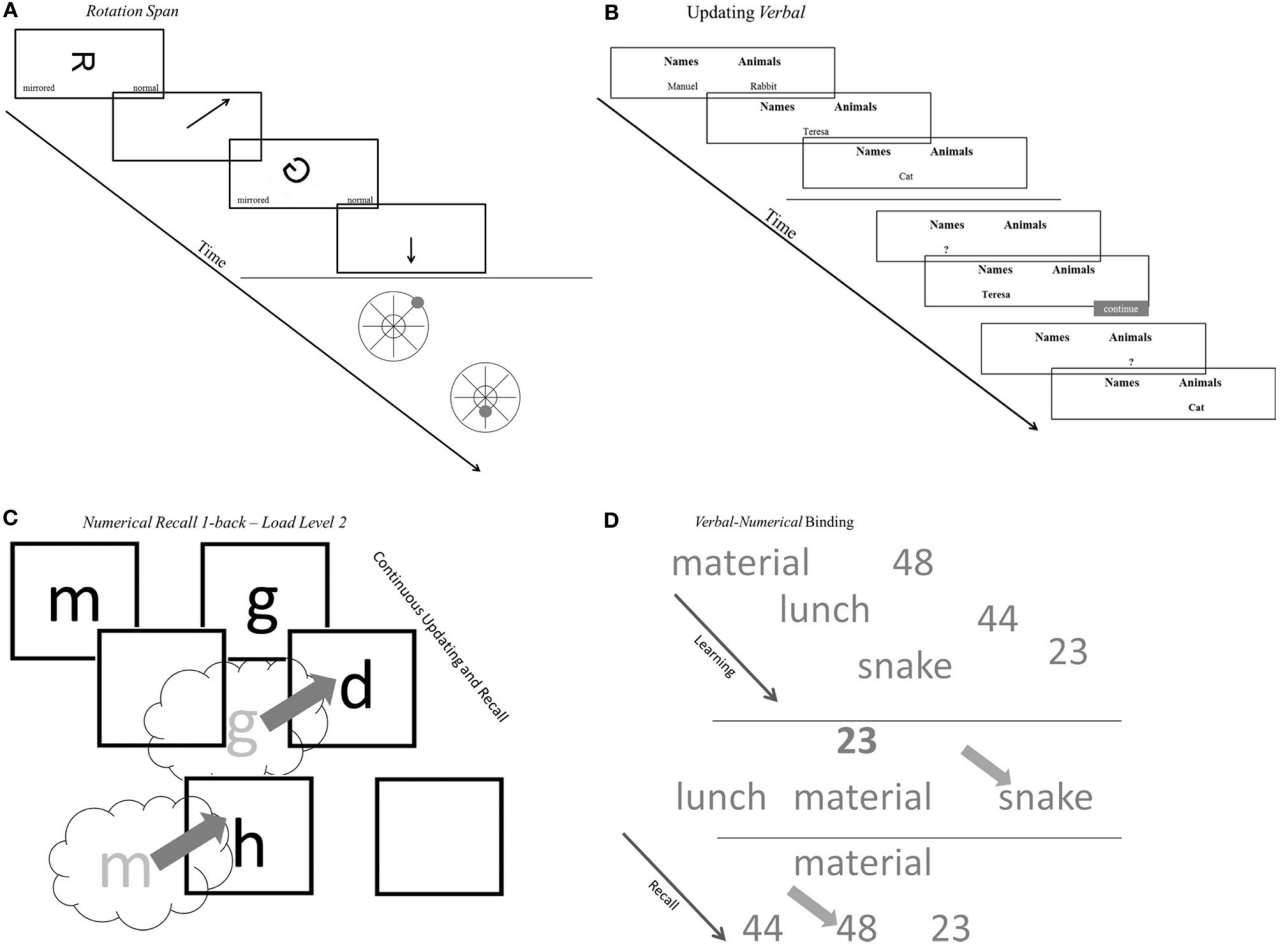
**Schematic representations of tasks. (A)** Schematic representations of complex span tasks (rotation span). **(B)** Schematic representations of updating tasks (updating verbal). **(C)** Schematic representations of recall 1-back tasks (numerical recall 1-back). **(D)** Schematic representations of binding tasks (verbal-numerical binding). See task descriptions for details.

The measures are conceptualized as indicators for four task classes. Arguably these task classes tap different aspects of the working memory system. Within each task class indicators relied on either verbal, numerical, or figural-spatial stimuli and responses, or mixtures of two of these content domains. The selected task classes reflect operationalizations of competing WMC accounts and are frequently used measures for the assessment of WMC. Based on these two criteria, four WMC task classes were identified: (1) *Complex Span* (Cspan) tasks designed to capture simultaneous storage and processing in conditions of high interference, (2) *Updating* tasks assessing the accuracy of updating in working memory across a series of steps, (3) *Recall N-back* (RNb) tasks requiring the evaluation of the identity of each stimulus from a sequence to a preceding stimulus presented with a certain lag *N*, which also captures the updating of temporary bindings in working memory and (4) *Binding* tasks developed to test the ability to establish and briefly maintain bindings in working memory (see Figure 2 for a schematic representation of an example task for the four task classes).

Besides these four classes for measuring WMC, three covariates were of relevance in the present study. First, we developed three tests for the assessment of SM, capturing the aptitude to establish new associations in memory. These tasks were designed analogous to the binding tasks, except that list lengths were longer in order to exceed the capacity limits of primary memory and that memory for associations was tested over a much longer retention period filled with other tasks to ensure that recall relies entirely on SM. Second, two popular experimental paradigms—the Simon task and the Eriksen flanker task—were included to measure one aspect of cognitive control, the inhibition of strong but wrong response tendencies. Finally, we administered three tests of fluid intelligence as criterion measures.

#### Complex span tasks (Cspan)

In the *reading span task*, adapted from Kane et al. (2004), participants recalled letters in the context of a simple reading comprehension task. Several sentences were presented successively on the screen. Below each sentence, a single letter was displayed simultaneously with the sentence. Participants had to memorize the letters. Additionally, they evaluated the meaningfulness of the sentences (e.g., “The police stopped Andreas because he crossed the sky at red light.” would require a “no” response). After assessing the meaningfulness of the last sentence of each trial, subjects recalled the letters of that trial in their order of presentation. Sentence-letter combinations were presented without time limits during learning and recall. The next stimulus appeared as soon as participants provided their response to the actual stimulus pair.

The procedure of the *operation span* task was very similar and applied the same conditions of presentation. Participants evaluated the correctness of arithmetic equations of low difficulty and memorized short words displayed simultaneously with the equations. The task requirement was to recall all words presented in a trial in the correct serial order after evaluating the last arithmetic equation.

The *rotation span* task—developed on the basis of an idea from Shah and Miyake (1996)—was also adapted from Kane et al. (2004). Participants recalled a sequence of short and long arrows radiating out from the center of the screen pointing into one of eight possible directions. They had to maintain this sequence while processing a figural verification task after each arrow. Each display presented a normal or mirror-reversed G, F, or R in the middle of the screen. Each letter was rotated by an angle selected at random from 8 possible angles, equally spaced by 45 degree steps. The participant's task was to decide whether the orientation of a letter was normal or mirror-reversed. After completing all elements of a trial, participants recalled all of the arrows of that trial in the sequence of their appearance. For recall a graphic depicting the 16 possible arrows appeared. Participants used the mouse to indicate the arrows they memorized by clicking on the corresponding points of the answer screen.

Load levels were two to five and we administered three practice and 12 test trials for each task, three for each load level (see Table A1 for details). The score for each trial was the proportions of list elements recalled in their correct serial positions. We use the average score across all trials of each task as indicators in the latent variable models (Conway et al., 2005). Descriptive results and reliability estimates—which were satisfactory to high, ranging between 0.74 and 0.87—are summarized in Table A2.

#### Updating tasks (updating) (e.g., Miyake et al., 2000)

The *verbal* updating task included 12 trials. Each trial used stimuli from 2 to 5 semantic categories, depending on the load level of the trial. Categories were *forenames*, *animals*, *fruits & vegetables*, *objects*, and *countries*. Words were presented one at a time for 2000 ms during trials of the load level 2, 2400 ms for load level 3, 2800 ms for load level 4 and 3000 ms for the highest load level respectively. After an Inter Stimulus Interval (ISI) of 500 ms the next word was presented. Participants' task was to keep the last word for each category in mind. After a variable and unpredictable number of updating steps (range 2–6) participants were asked to type the last word presented for each category.

In the *numerical updating* task digits were displayed in 2–6 boxes (depending on the load-level of a trial). A variable and unpredictable series of digits per box (range 2–6) was presented one by one for 1600 ms per digit. Each digit was displayed in a box selected at random. Subjects were asked to keep in mind the last digit for each box, and type the digits for each box at the end of each trial. We administered 12 numerical updating trials.

In the *spatial-figural updating* task rectangles of different colors were presented on different positions within a 3 × 3 grid matrix. Depending on the load level of a trial, we used two to five different colors. Colored rectangles were presented one at a time at a rate of 2000 ms presentation at load level 2, 2400 ms at load level 3, and 2800 ms at load level 4 and 5. The ISI was 500 ms. The number of positions for each rectangle in different trials ranged from 2 to 5 and was unpredictable. Subjects were asked to keep track of the last position of each color. At recall, rectangles of the different colors used in the current trial were presented one at a time under the grid, and participants responded by clicking with the mouse into the grid cell where the target color appeared last.

Trial scores are the proportion of correctly recalled words, numbers, or color positions, respectively. We use the proportion of correct responses across all trials of a task as dependent variables in the models. Table A1 displays the detailed trial design. Table A2 shows descriptive statistics and reliability estimates for all task versions. Cronbach's α and McDonald's ω —computed as a reliability estimate for the latent variable in unidimensional measurement models across all trials of a task version—were satisfactory to good.

#### Recall 1-back (RNb) (Dobbs and Rule, 1989)

**Figure 3:**
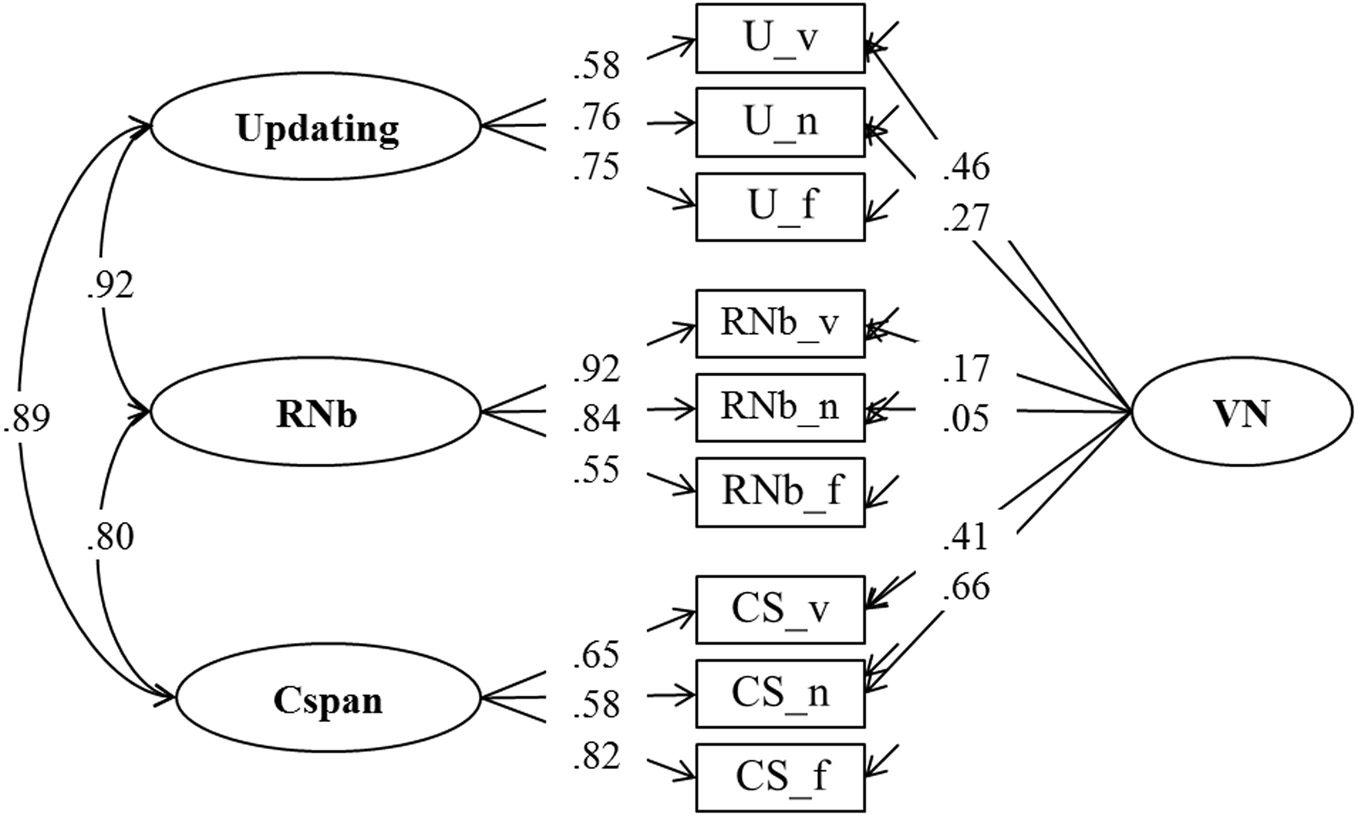
**Confirmatory factor analysis of updating, Recall 1-back (RNb) and Complex Span (Cspan) Tasks (Model 1).** The model postulates a verbal-numerical nested factor (VN that is orthogonal to task class specific factors). _v, verbal indicators; _n, numerical indicators; _f, figural indicators.

Three 1-back tasks were designed to measure recall of continuously updated elements. The tasks were similar to the updating tests used by Schmiedek et al. (2010). In the *verbal* RNb task, letters were presented one by one in 1 to 3 boxes, depending on the load level of the trial; see Table A1 for details. Each time a new letter appeared in a box, participants typed the last letter that has been presented previously in that box. Each response had to be completed within the time for which the new letter was presented; responses after this interval were counted as errors. The procedure was equivalent in the *numerical* RNb task. Digits were presented instead of letters in the boxes, and participants responded by typing the last digit that had been presented previously in a given box. In the *spatial-figural* RNb task different abstract Figures (1–3, depending on the load level) were displayed in randomly selected cells of a 3 × 3 grid. For every new figure presented, participants had to indicate the last location in the grid in which the respective figure has been presented one step before. Participants responded by clicking in the correct grid cell with the mouse; they had to respond as long as the figure was presented. The presentation intervals for individual stimuli, which determine the response windows, varied across RNb tasks and were based on results from pilot studies. In the verbal task, intervals were 2500 ms at load 1, 3000 ms at load 2, and 3500 ms at load 3. In the numerical RNb task response intervals were 2500 ms at load 1, 2900 ms at load 2, and 3100 ms at load 3. In the spatial-figural RNb task presentations intervals were 2500 ms at load 1, 3500 ms at load 2, and 4500 ms at load 3. More specifics concerning the task design is provided in Table A1 and Figure 2 shows a schematic representation of the trial sequence.

Proportion of correct responses across queries in a trial was defined as score. The mean across trials formed the task scores that served as dependent variables in the latent variable models. Descriptive statistics and reliability estimates—which were all excellent (α and ω all above 0.90)—are provided in Table A2.

#### Binding tasks (binding)

The binding tests relied on pairing stimuli from different content domains. Therefore, the assignment of tests to content domains is less clear than for other task classes. In the *letter-color binding* task, each trial involved sequential presentation of a short list of letter-color pairs that participants had to remember. This was followed immediately by a recall test, probing each pair in a random order. In one half of the tests the color of a pair was presented centrally, and all letters used in the current trial were lined up horizontally below. Participants had to select the letter that had been paired with the given color. For the other half of the trials the color that had been paired with a given letter had to be selected from a list of all colors presented in the current trial. Thus, the test made no requirement on item memory (i.e., remembering which letters and which colors were used in the current trial) but tested only memory for their pairwise relations, which requires maintenance of temporary bindings. To increase the demand on temporary bindings in WM as opposed to associations in long-term memory, the binding tests used a small set of letters and colors repeatedly throughout all trials, so that across successive trials the same letters and colors were paired in many different ways. Accuracy therefore depended on remembering the pairing in the current trial as opposed to different pairings of the same elements in previous trials.

In the *word-number* binding task participants remembered several pairs of nouns with two-digit numbers. Recall was tested as in the letter-color task. In the *location-letter* binding task participants were asked to remember the positions of letters within a 3 × 3 grid. During recall participants had to indicate the location of a given letter, or select the letter that was displayed at a given location in the grid.

Load levels ranged from 2 to 6 pairs in each of the three binding tasks. There were 15 trials for the letter-color task and 14 trials for the two other binding measures. The number of trials per load level is provided in Table A1. In the letter-color task presentation time of each pair was 1000 ms, and the ISI was 3000 ms. In the word-number task presentation time was 2000 ms, followed by an ISI of 1000 ms. In the location-letter test stimuli were presented for 1500 ms, and the ISI was 500 ms. There was no response deadline at recall.

Trial scores were the proportion of correctly recalled pairs. Average performance across all trials in each task served as the indicator analyzed in latent variable models. Descriptive statistics and reliability estimates of these indicators are provided in Table A2. Internal consistency (Cronbach's α) was 0.69 for the letter-color, 0.79 for the word-number and 0.79 for the location-letter version of the task and McDonald's ω was satisfactory for all three indicators.

#### Secondary memory tasks (SM)

The SM tasks were constructed in close analogy to the binding measures. In the *word-word* SM task participants learned 20 word pairs presented sequentially. In the *word-number* task participants learned a sequentially presented list of 20 pairs of one word and one two-digit number each. In the *letter-position* SM task participants had to learn 12 associations of letters to positions within a 4 × 4 grid.

Presentation time for each stimulus pair was 4000 ms, followed by ISI of 1000 ms for all SM tests. There were two blocks of trials, each consisting of 20 pairs in the word-word and the word-number task, and 12 pairs in the letter-position task, respectively. The two blocks included different lists of stimuli, and they were located at different positions within the testing sessions (see Table A1 for details).

Between encoding and recall subjects completed other tasks to ensure that they could not retain some of the pairs in working memory. The tasks completed in this retention interval lasted ~3 min, and they tested either mental speed or cognitive control. There was no time limit at recall. In the word-word task participants were provided with either the first or second word from a pair and had to type in the missing word. In the word-number tasks participants received either the word or the number and had to type in the missing associate. In the letter-position task participants indicated the position of a given letter in the grid, or the letter that was presented on a given position.

Trial scores were the proportion of correctly recalled pairs within a block. The average performance in the two blocks of a task was then used as dependent variable for the latent variable models. Reliability estimates were satisfactory (see Table A2 for descriptive statistics and reliabilities).

#### Reasoning tasks (Gf)

In line with the variation of content domains in the WMC measures, assessment of reasoning ability was based upon three tests varying in content. We administrated the fluid intelligence section of the *Berlin Test of Fluid and Crystallized Intelligence* (BEFKI; Wilhelm et al., 2013). Participants were asked to deduce valid conclusions from a set of premises in the verbal version. In the numerical version participants had to solve arithmetic reasoning problems. In the figural subtest participants had to infer regularities in series of geometric figures that changed their shape, position, and shading. Based upon this inference participants had to select two further elements that correctly completed the sequence.

Reasoning measures were paper-pencil based and had multiple-choice format. There were 16 items in each test and 14 min of testing time per domain. Scores and dependent variables for the models were the proportion of correctly solved items for each content domain. Descriptive statistics and reliability estimates for the three indicators are given in Table A2.

#### Tasks measuring response inhibition (inhibition)

Stimuli for the *Eriksen Flanker* (E) task were five left or right pointing arrows presented in a row in the center of the screen. Participants were asked to indicate the direction of the third (middle) arrow by pressing the corresponding arrow keys—left (number 4) or right (number 6)—on the number block of a regular keyboard. Answer keys were labeled by colored stickers. Flanker arrows all pointed to the same direction. The flanker direction was either the same as that of the target arrow (compatible task condition) or the opposite direction (incompatible task condition).

We used diamonds and squares as stimuli for the *Simon* (S) task. These stimuli had a size of approximately 30 × 30 mm and were presented below or above a central fixation cross. Participants were asked to respond by pressing the key with the upright pointing arrow on the regular keyboard (labeled with a sticker) when they saw a diamond, and the key with the down pointing arrow when they saw a square. Thus, a diamond in the upper half of the screen, or a square in the lower half, were compatible trials, whereas a diamond in the lower half and a square in the upper half were incompatible trials (Stürmer et al., 2002).

Both tasks were applied as intermediate tasks in the retention interval of the SM measures. There were two blocks for both measures, each including 80 trials (half of which were incompatible). Trials were classified according to three independent variables (cf. Keye et al., 2009): Congruency of the trial itself, congruency of the preceding trial, and repetition priming (i.e., whether or not the stimulus of the current trial was identical or not to the stimulus of the preceding trial); crossing of these three variables resulted in eight conditions. Dependent variables were averages of the inverted latencies obtained across all correct responses of each condition. Inverted latencies were calculated as 1,000 divided by the reaction time (RT) in milliseconds. Descriptive statistics and reliability estimates are displayed in Table A2.

### Data treatment

Both test sessions were attended by 267 participants. Five of them had missing values on more than four tasks due to technical problems during testing and were excluded from the sample. As a first step of data cleaning we visually screened the univariate distributions for each performance indicator. Observations indicative of floor effects were set to missing value. Outlier values—defined as observations that were located above the whiskers of the boxplots (*g* = 1.5) of the univariate distributions—were set to missing (Tukey, 1977). With the present sample size the applied settings of this outlier labeling rule are adequate (Hoaglin et al., 1986). Across all indicators a total of 34 scores (0.5% of the data) were missing, including the outlier scores that have been set to missing. There were no missing data for half of the 34 indicators considered in this paper. We performed a multiple random imputation (e.g., Allison, 2001; Sinharay et al., 2001) to generate 20 datasets, differing only in the imputed values. These data sets are the basis of subsequent analysis. Based upon this procedure of handling missing data the variability of the imputed plausible values across multiple datasets can be used to adjust the standard errors (SE) of the parameter estimates in the structural equation models. Plausible values are computed as predicted values for the missing observations plus a random draw from the residual normal distribution of the respective variable. The imputation was carried out based on a multiple regression under the multivariate normal model that included all other indicators as predictors in the imputation model. Although the multivariate normal model implies strong assumptions—specifically normally distributed variables and normal and homoscedastic error terms—Schafer (1997) showed that the normal model performs well even for variables that are not normally distributed at the manifest level, especially if non-normal distributions are observed for variables without missing values. In our case the normality assumption was violated for 6 out of 17 variables with missing data. In order to overcome possible drawbacks due to these violated assumptions and investigate the robustness of the results we will also report bootstrapped parameter estimates computed for a single imputed dataset.

In a second step of initial data screening, we estimated a confirmatory factor model for the core constructs investigated in the present study and inspected Mahalanobis distances of participants as a measure of deviance in the multivariate distribution. There were no observations standing out from the rest of the sample, as indicated by their Mahalanobis-values. Therefore, no further outlier correction was required.

### Statistical analyses

All Confirmatory Factor Analyses (CFA) and Structural Equation Models (SEM) were run in *Mplus* 6 (Muthén and Muthén, 1998–2010) using two different estimation procedures: (1) multiple imputation procedures implemented in *Mplus* (Asparouhov and Muthen, 2010) and (2) Maximum Likelihood (ML) estimation with bootstrap SEs and confidence intervals (CIs) of the model parameter and bootstraped *p*-value for the χ^2^ statistics.^4^ The latter were obtained using the residual bootstrapping option implemented in *Mplus*, which corresponds to the Bollen-Stine bootstrap procedure (Bollen and Stine, 1992). ML estimation was computed on the basis of a single dataset. For all models 1,000 bootstrap samples were drawn. We report confidence intervals that are based upon this bootstrap procedure. For the analysis based on multiple datasets, parameter estimates are averaged over the set of analyses by the program. The SE are computed using the average of the SE and the estimated parameter variation between analyses using the formula of Rubin (1987). *Mplus* provides a chi-square test of overall model fit for analysis based upon imputed data (Asparouhov and Muthen, 2010).

A variety of indices are commonly used to assess the adequacy of structural equation models (Bollen and Long, 1993). An important statistic is the χ^2^-value that expresses how similar the model-implied covariance matrix and the observed covariance matrix are. Higher values indicate stronger deviations of the observed from the implied covariance matrix. For many purposes the χ^2^-test is not optimal because its power heavily depends on sample size. Therefore, we will report additional fit indices. The Root Mean Square Error of Approximation (RMSEA) is an estimate of misfit due to model misspecification per degree of freedom. The Standardized Root Mean-square Residual (SRMR) reflects the standardized difference between observed and the model-implied covariance matrix. The Comparative Fit Index (CFI) is known as an incremental fit index that expresses the proportion of improvement in overall fit relative to a model assuming no correlations between the manifest variables (independence model). Together these statistics and indices allow the assessment and evaluation of model fit. Some cut-off values have been established as rules of thumb for evaluating the fit on the bases of these indices. If the sample size is not large, the χ^2^-statistic should not surpass the conventional level of significance. Regardless of sample size the CFI values should be 0.95 or higher, RMSEA values should be 0.06 or smaller, and SRMR values should be 0.08 or smaller (Hu and Bentler, 1995).

## Results

We tested a series of latent variable models to address the research questions outlined above. In the first section we investigate the scope of the WMC construct and show that WMC encompasses both complex-span and updating tasks. In the second section we test the prediction of the binding hypothesis that WMC also encompasses a test of binding memory. In the third section we test the hypothesis of the two-component theory of a close relationship between WMC measures—in particular Complex Span—and SM. The fourth section tests the hypothesis from the executive-attention theory that Inhibition is closely related to WMC. In the final section we investigate the relation of WMC and SM to fluid intelligence.

### Complex span, recall 1-back and updating tasks

As can be seen in Figure 1, all three theories under consideration agree that complex-span tasks and updating tasks share a substantial proportion of their variance, although they suggest different interpretations of this shared variance: According to the executive-attention theory it reflects variance in general executive attention; according to the two-component theory it reflects primarily PM, and according to the binding hypothesis it reflects the binding ability assumed to underlie WMC. In addition, the two-component theory motivates the prediction that the correlation between task classes varies as a function of SM contribution to task performance. Arguably, for complex span the SM contribution is larger than for Recall N-back and Updating. Therefore, the latter two should be more highly correlated among each other than each of them is correlated with Cspan. To test these hypotheses we estimated *Model 1* as a confirmatory factor model with four latent factors.

Model 1, presented in Figure 3, postulates three task factors, one for each task class (Cspan, RNb, and Updating). Additionally, a fourth content specific factor is included to account for individual differences due to the verbal-numerical content used in six of the nine indicators. The content factor is specified as being orthogonal to the three task factors. The task factors are estimated as being correlated. Model fit estimated on the basis of the multiple datasets was excellent: χ^2^[18] = 31.68, *p* = 0.024, CFI = 0.989, RMSEA = 0.054, SRMR = 0.025 (the bootstrap *p*-value for the χ^2^-test computed for a single dataset was 0.138). All factor loadings on task factors were large and significantly different from zero. They are given in Figure 3 and in Table 1 together with bootstrap estimates of the 95% CIs computed on a single dataset. Loadings on the verbal-numerical content factor (VN) were lower, but still substantial and significantly larger than zero. The correlations between task factors were *r* = 0.89 between Cspan and Updating (single dataset for bootstrapped estimates *r* = 0.90), *r* = 0.80 between Cspan and RNb (single dataset 0.80), and *r* = 0.92 between Updating and RNb (single dataset 0.92). These very high correlations confirm that the Updating and RNb task classes designed for this study are essentially measuring the same construct. Furthermore, they are also highly related with Cspan. We compared Model 1 with a simplified Model 1 b in which a single WMC factor replaces the three task factors. The fit of this general-factor WMC model was poorer than the fit of Model 1: χ^2^[21] = 53.18, *p* < 0.01, CFI = 0.974, RMSEA = 0.076, SRMR = 0.032.

**Table 1:** **Estimates of Loadings for Model 1 (and Bootstrap Estimates of the 95% Confidence Intervals based on a Single Dataset) and Standardized Loadings (and Standard Errors Estimated on Multiple Datasets)**.

	**Verbal indicators**		**Numerical indicators**		**Figural indicators**	
	λ	λ **(std)**	λ	λ **(std)**	λ	λ **(std)**
Updating	0.09 (0.07–0.12)	0.58 (0.05)	0.13 (0.10–0.15)	0.76 (0.04)	0.11 (0.09–0.13)	0.75 (0.03)
RNb	0.18 (0.16–0.20)	0.92 (0.02)	0.17 (0.14–0.20)	0.84 (0.02)	0.09 (0.07–0.10)	0.55 (0.05)
Cspan	0.13 (0.10–0.15)	0.65 (0.05)	0.08 (0.06–0.09)	0.58 (0.05)	0.15 (0.12–0.17)	0.82 (0.04)

Note: RNb, Recall 1-back; Cspan, Complex Span; λ, factor loading; std, standardized.

### Binding and working memory capacity

Concerning the relation between the first three working-memory task classes (Cspan, Updating, & RNb) and the binding measures, the three theories make partly different predictions. From the executive-attention view, binding tasks are not different from short-term memory measures such as digit span and word span, which are assumed to require less control of attention than complex-span tasks and updating tasks. Therefore, the binding tasks should correlate less with the other three WMC task classes than these task classes correlate with each other. According to the two-component theory, correlations between tasks should reflect the relative contributions of SM and PM to each of them. Because in the binding tasks, short lists of pairs are presented briefly without intervening distraction, there is little time to encode them into SM. Moreover, the use of a small set of elements re-paired in each trial would create massive proactive interference in SM, so that reliance on SM is unlikely to be helpful. In this regard, the binding tasks are similar to the updating tasks that likewise must rely mostly on PM. Therefore, binding tasks should be more closely correlated with the updating tasks than with complex span, which arguably relies to a larger extent on SM.

**Figure 4:**
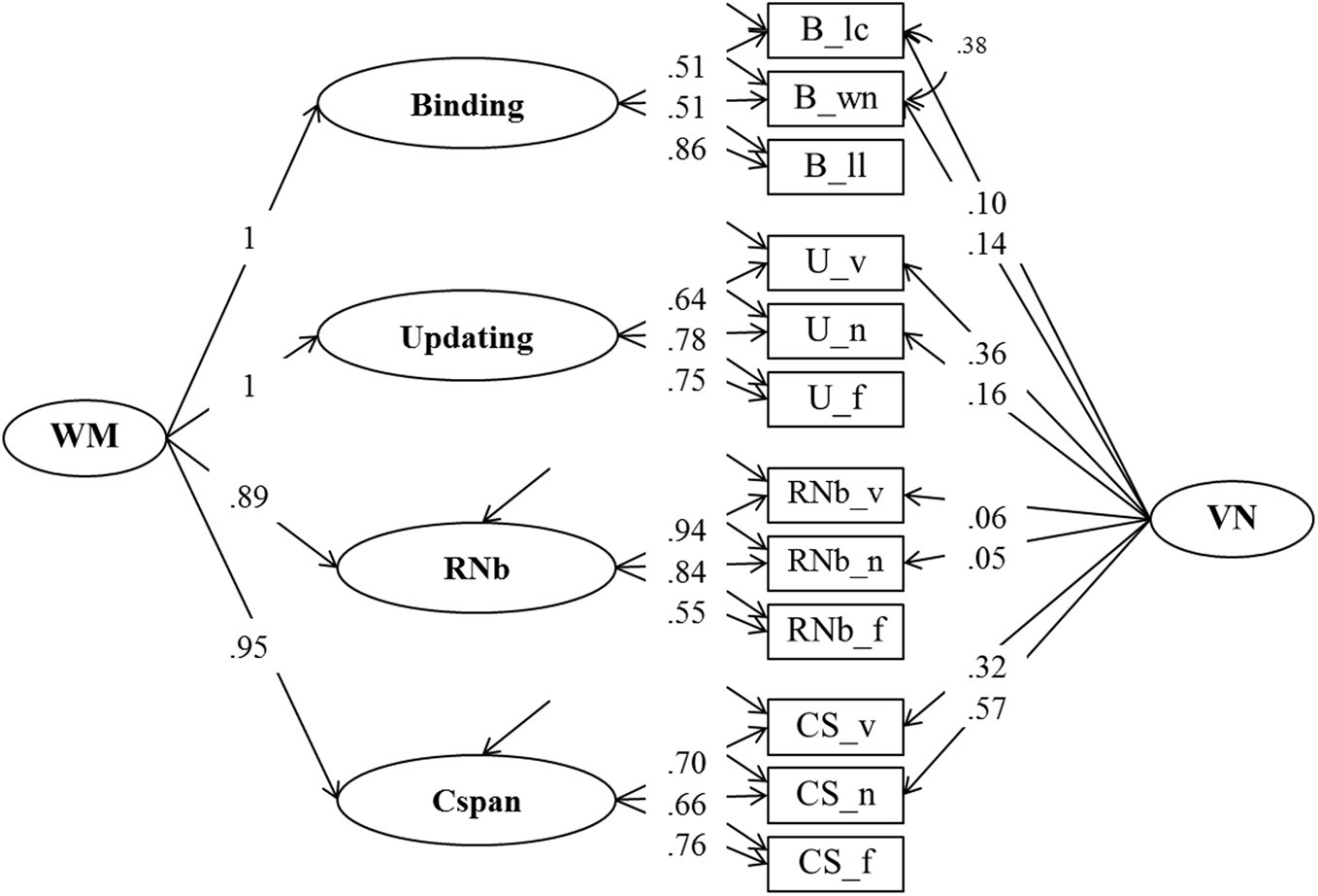
**Confirmatory factor analysis of Binding, Updating, Recall 1-back (RNb) and Complex Span (Cspan) Tasks (Model 2).** The common variance of the task factors is accounted for by the higher-order Working Memory (WM) factor. The loadings of the Binding and Updating factors on WM were set to 1, the disturbance term of these factors was constrained to zero. _v, verbal indicators; _n, numerical indicators; _f, figural indicators; _lc, letter-color; _wn, word-number; _ll, location-letter; CS, Cspan indicators; VN, Verbal-numerical content factor.

From the perspective of our binding hypothesis of WMC, all four task classes (Cspan, Updating, RNb, and Binding) primarily reflect individual differences in the ability to quickly build, briefly maintain, and rapidly update arbitrary bindings. Therefore, they should all be highly if not perfectly correlated. In order to establish the structure of individual differences across all four WMC task classes, Model 1 was extended with an additional factor for the binding tasks. The additional factor had to account for the shared variance of the three binding indicators (letter-color, word-number, and location-letter). Initially, the factor intercorrelations for the four task factors were freely estimated. The correlations among all four task factors were found to be very high. Therefore, we replaced them by a higher-order factor model (*Model 2*)—depicted in Figure 4—as the final representation of the WMC construct to be used in further analyses. Freely estimated standardized loadings of the Binding and Updating factors on the second order WMC factor did not differ significantly from one. Thus, in the final solution for *Model 2* these factor loadings were set to one, and the residual variance of the two first order factors was constrained to zero. RNb and Cspan loadings were also very high: 0.89 and 0.95 respectively.

The fit of Model 2 was very good: χ^2^[43] = 74.47, *p* = 0.002, CFI = 0.982, RMSEA = 0.053, SRMR = 0.030 (bootstrap *p*-value for the χ^2^-test computed for a single dataset was 0.074). Loadings on the first-order factors (Updating, RNb, Cspan and Verbal-Numerical) did not substantially differ from those from Model 1. Standardized loadings on the Binding factor ranged between 0.51 and 0.86, with a highest loading of the letter-position task. Verbal-numerical content-specific variance was accounted for by the VN factor that was introduced in Model 1 and also had loadings from the letter-color and the word-number binding tasks. There was a substantial residual covariance (0.38) between the verbal and numerical Binding indicators, which we interpret as shared method variance of learning pairwise relations within the verbal-numerical content domain.

### Working memory capacity and secondary memory

We next address the relation between the general WMC factor established in Model 2 and SM. According to the view that complex span reflects to a substantial extent the efficiency of search in SM, a strong relation with the measures for SM ought to be predicted. The relation of SM indicators with the other WMC task classes is expected to be lower, because these task classes rely less on SM. In contrast, the executive-attention view of WMC provides no reason to expect a high correlation between WMC and SM. Finally, according to the binding hypothesis, we should expect that maintaining robust bindings in WM facilitates long-term learning of those bindings. Therefore, we should expect a positive correlation between WMC and SM on the level of general WMC, not specifically for Cspan.

**Figure 5:**
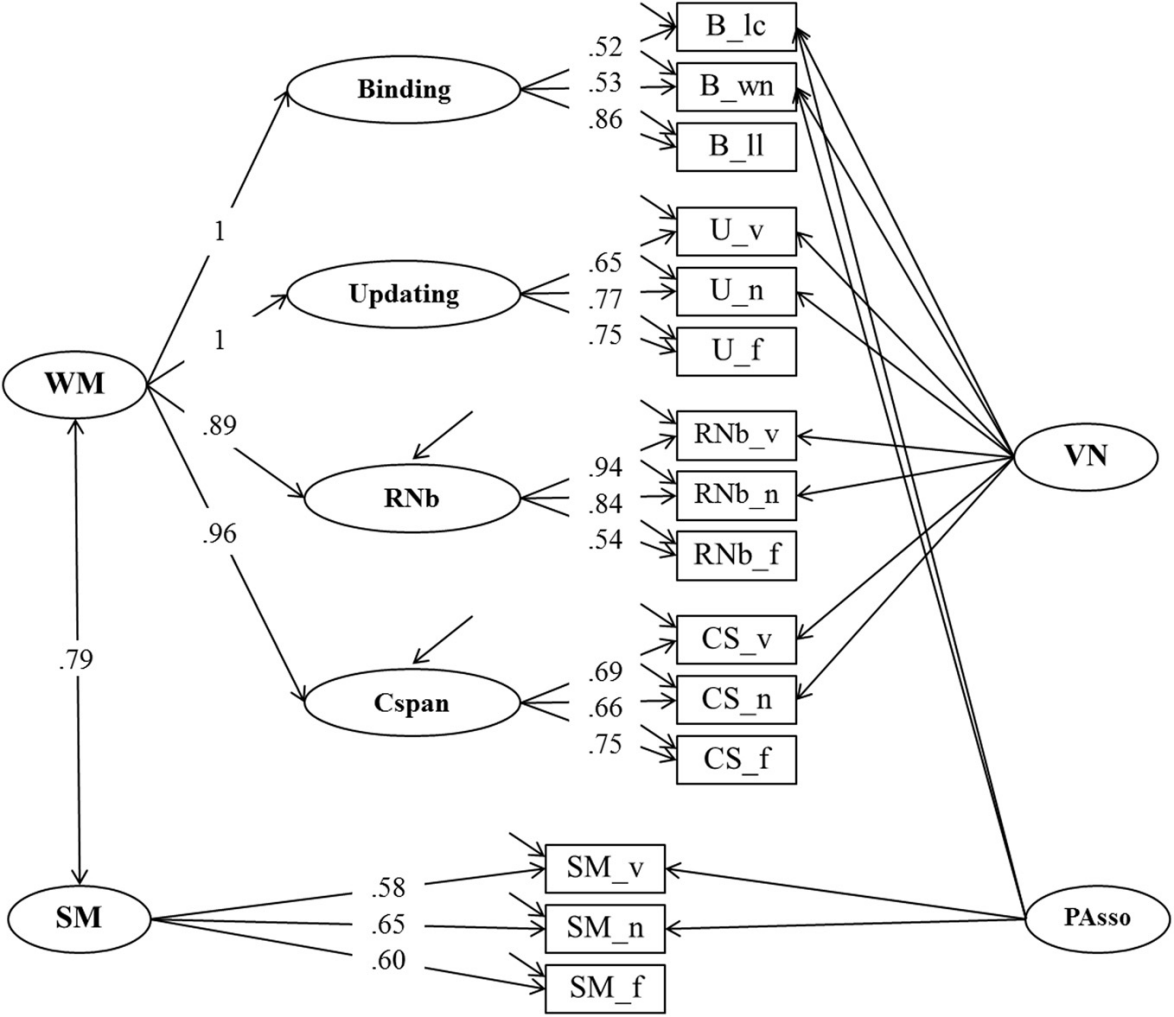
**Structural equation model of WMC and Secondary Memory (SM) (Model 3).** The measurement model of WMC is identical with the one depicted in Figure 3, postulating a higher order model for WMC with four first order factors: Binding, Updating, Recall 1-back (RNb) and Complex Span (Cspan). The VN (Verbal-Numerical Content) factor is maintained from Model 2. SM, Secondary Memory; PAsso, Learning Paired-Associations; _v, verbal indicators; _n, numerical indicators; _f, figural indicators; _lc, letter-color; _wn, word-number; _ll, location-letter; CS, Cspan indicators.

To test the relationship of WMC and SM we extended Model 2 by adding an SM factor that accounts for common variance among the three SM tasks (*Model 3* depicted in Figure 5). Additionally, Model 3 introduces a second nested factor *PAsso*. This factor was introduced to capture method variance due to paired-associates learning with verbal or verbal-numerical content. This method factor was foreshadowed in Model 2 by the residual covariance between the letter-color and the word-number binding tasks.

*Model 3* had a very good fit: χ^2^[75] = 117.07, *p* = 0.001, CFI = 0.981, RMSEA = 0.046, SRMR = 0.034 (bootstrap *p*-value for the χ^2^-test computed for a single dataset was 0.044). Loadings of task scores on the SM factor were all substantial and ranged between 0.58 and 0.65. Verbal-numerical Binding and SM indicators loaded substantially and significantly on the *PAsso* factor (0.39–0.71). The latent level correlation between the second-order WMC factor and SM was 0.79 (bootstrap estimates of the 95% CI based on a single dataset was 0.70–0.87), and statistically different from unity (Δ χ ^2^[1] = 22.86). This result implies that WMC and SM are closely related but not identical constructs. Additionally estimating the relation between the first order residual term of the Cspan factor and SM did not affect model fit which implies that this relation was not reliably different from zero (χ^2^[74] = 115.16, *p* = 0.002, CFI = 0.982, RMSEA = 0.046, SRMR = 0.034; compared with Model 3 Δχ ^2^[1] = 1.91, *p* = 0.17).

### Working memory capacity and controlled attention

We next explored associations of WMC with measures of cognitive control, in particular response inhibition in the Eriksen flanker and the Simon paradigm. The executive-attention view of WMC (Engle et al., 1999; Engle, 2002; Engle and Kane, 2004) predicts a substantial positive correlation between WMC and the success of overcoming cognitive conflict through the inhibition of strong but wrong response tendencies. The success of response inhibition can be gauged through individual differences in conflict effects (i.e., the size of the Simon congruency effect and of the Eriksen flanker congruency effect). To test the prediction of the executive-attention view we first needed to establish a measurement model for the two conflict paradigms to obtain a measure of the success of response inhibition.

#### Measurement model of response inhibition

**Figure 6:**
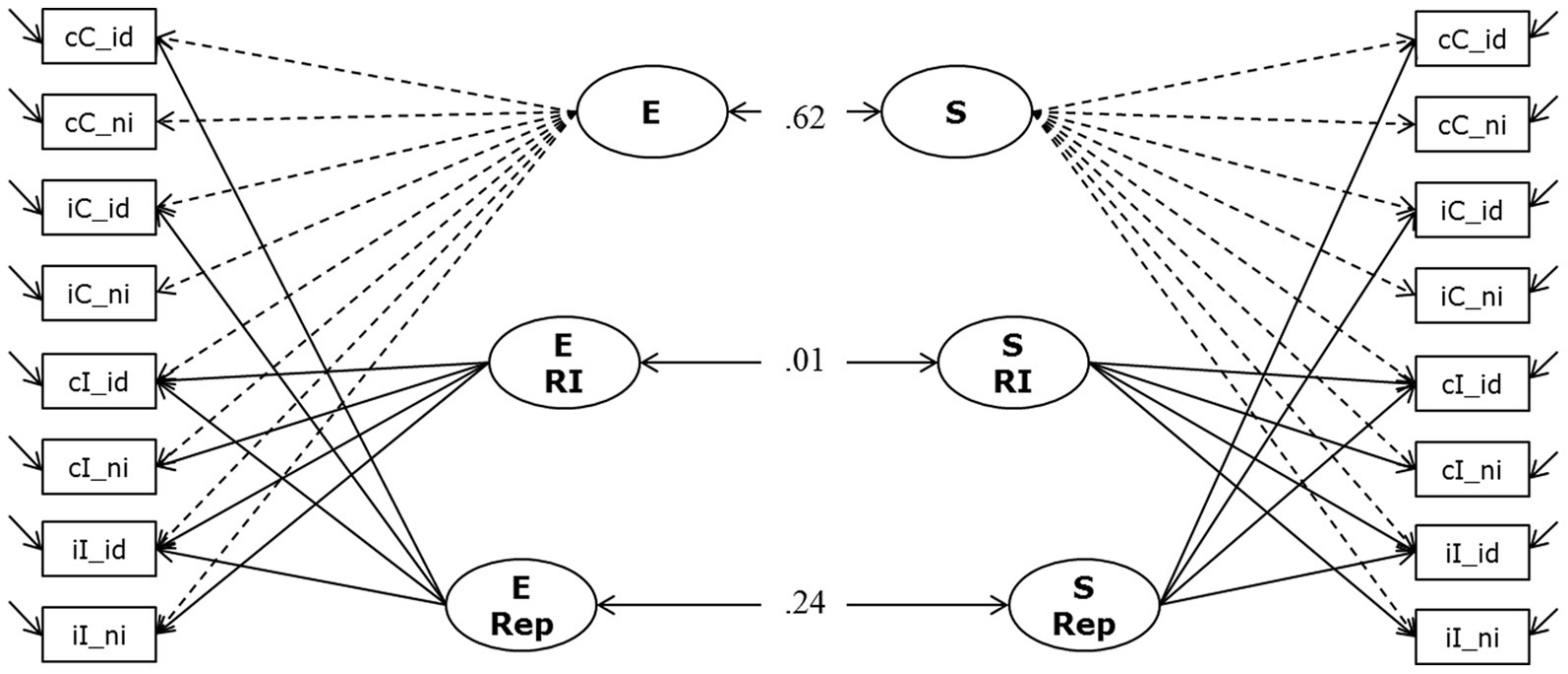
**Measurement Model of Response Inhibition (Model 4a).** The model postulates two general factors (E and S); which account for individual variation in the number of correctly solved items per second (inverted latencies) during the Eriksen Flanker task and the Simon task, respectively. Additionally, for each paradigm we specified a factor to capture variation in response inhibition, and another factor for repetition priming (E RI, E Rep, and S RI and S Rep). Indicators were built to reflect sequence modulations of performance; cC, compatible trials following compatible trials; iC, compatible trials preceded by incompatible trials; cI, incompatible trials following compatible trials; iI, incompatible trials preceded by incompatible trials; _id, identical (stimulus repetition, relative to preceding trial); _ni, non-identical (stimulus change, relative to preceding trial).

To investigate whether the two paradigms (Simon and Eriksen flanker) are measuring the same underlying construct of response inhibition we specified a measurement model relating latent factors of performance on the Simon task and the Eriksen Flanker task. The measurement model (*Model 4a*) is depicted in Figure 6. According to our previous experience with the modeling of individual differences in these response-conflict paradigms (Keye et al., 2009, 2010) we postulated three factors for each paradigm. Besides a general factor representing general efficiency in each paradigm we specified a conflict factor (CC-E and CC-S, respectively) to account for individual differences in the size of the congruency effects in each paradigm. Additionally, we specified a repetition-priming factor to capture individual differences in the size of the priming effect in cases of a perfect match between the previous and current stimuli in the Simon and Eriksen tasks (E-Rep and S-Rep respectively). We allowed for correlations between corresponding factors across paradigms.

The model depicted in Figure 6 fitted the data acceptably well: χ^2^[86] = 330.91, *p* = 0.000, CFI = 0. 947, RMSEA = 0.104, SRMR = 0.046 (bootstrap *p*-value for the χ^2^-test computed on a single dataset was 0.05; χ^2^[86] = 341.24). We attribute the rather high RMSEA statistic to the high average zero-order correlation and the high communalities in this analysis. Prior research shows that small correlations of residuals—indicating a decent fit of the model—might come along with indications of bad fit in terms of conventional criteria when the unique variances are small (Browne et al., 2002; Heene et al., 2011). Standardized loadings on the general factors were high (all above 0.74), and standardized loadings on the specific experimental factors were lower (as expected for nested factors) but substantial. The model allows for correlations between corresponding factors for both paradigms. All other correlations were theoretically not expected and were fixed to zero. Models in which these fixed correlations between latent factors were estimated freely resulted in small estimates not surpassing the conventional significance criterion. There was a moderate to strong correlation between the two general factors (E and S), *r* = 0.62 (*p* < 0.001; bootstrap estimates of the 95% CI from 0.49 to 0.78). However, the two conflict factors were completely unrelated: estimated *r* = 0.008 when modeling multiple datasets. The bootstrap estimate of the 95% CI based on a single dataset ranged between −0.21 and 0.18. Repetition priming factors between task paradigms were weakly correlated (*r* = 0.24). Due to the lack of latent variable correlations for the two conflict factors—which are of focal interest in their relation with WMC—and in order to keep model complexity relatively low, the relationships of cognitive-conflict factors with WMC and SM were studied in two separate structural equation models (SEM), one investigating the correlation of conflict in the Simon task with WMC and SM, and the other doing the same for conflict in the Eriksen task.

#### Working memory, secondary memory and response inhibition in the simon task

**Figure 7:**
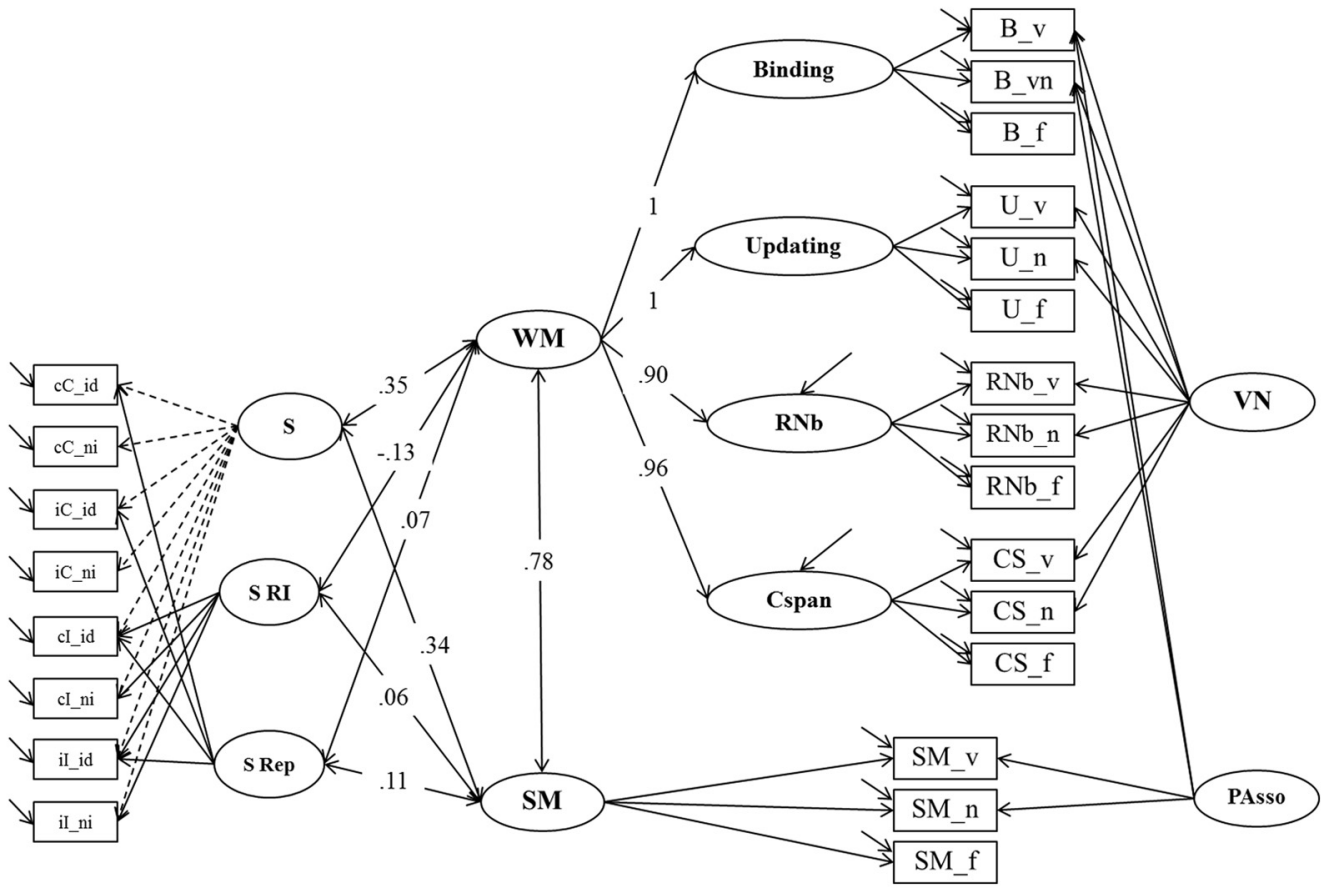
**Structural Equation Model testing the relationship of WM, SM and Response Inhibition un the Simon task (Model 4b).** Binding, Updating, Recall 1-back (RNb) and Complex Span (Cspan); VN, Verbal-Numerical Content Factor; SM, Secondary Memory; PAsso, Learning Paired-Associations; _v, verbal indicators; _n, numerical indicators; _f, figural indicators; _lc, letter-color; _wn, word-number; _ll, location-letter; CS, Cspan indicators; S, General RT performance during the Simon task; S RI, Response Inhibition effect in the Simon task, S Rep, Repetition Priming during the Simon task; cC, compatible trials following compatible trials; iC, compatible trials preceded by incompatible trials; cI, incompatible trials following compatible trials; iI, incompatible trials preceded by incompatible trials; _id, identical (Repetition Priming); _ni, non-identical (Unprimed).

In *Model 4b* (Figure 7), the sub-structure of Model 4a describing individual differences in the Simon task was related to Model 3. The fit of this model was acceptable: χ^2^[201] = 438.72, *p* = 0.000, CFI = 0.950, RMSEA = 0.067, SRMR = 0.053 (bootstrap *p*-value for the χ^2^-test computed on a single dataset was 0.016; χ^2^[201] = 385.95). Factor loadings did not notably differ from those estimated in the measurement models (Model 3 and Model 4a). There were 6 correlations of interest estimated in this model: The general factor from the Simon task (S) was moderately correlated with WM (*r* = 0.35; bootstrap estimate of the 95% CI: 0.22–0.48) and with SM (*r* = 0.34; bootstrap estimate of the 95% CI: 0.20–0.48). No significant correlation was observed between the specific conflict factor (S RI) and WM (*r* = −0.13; *p* = 0.36) and SM (*r* = 0.06; *p* = 0.74). WM and SM were also unrelated to Repetition Priming (WM: *r* = 0.07; *p* = 0.37; SM: *r* = 0.11; *p* = 0.19).

#### Working memory, secondary memory and response inhibition in the Eriksen flanker task

*Model 4c* had the same structure as Model 4b, except that the sub-structure of *Model 4a* describing individual differences in the Eriksen task was related to Model 3. This model had the same constraints on correlations as Model 4b. Model 4c fitted the data acceptably well: χ^2^[201] = 317.56, *p* < 0.001, CFI = 0.974, RMSEA = 0.047, SRMR = 0.044 (bootstrap *p*-value for the χ^2^-test computed on a single dataset was 0.198; χ^2^[201] = 324.60). Factor loadings did not notably differ from those estimated in the antecedent measurement models (Model 3 and Model 4a). The general factor reflecting overall performance on the Eriksen task (E) was moderately correlated with WM (*r* = 0.49; bootstrap estimate of the 95% CI: 0.35–0.63) and SM (*r* = 0.43; bootstrap estimate of the 95% CI: 0.29–0.57). Similarly to Model 4b the Eriksen Response Inhibition factor was not significantly related to WM (*r* = −0.12; *p* = 0.06) and SM (*r* = −0.08; *p* = 0.30). There was a small correlation between the repetition priming factor and WM (*r* = 0.16; *p* = 0.011; bootstrap estimate of the 95% CI: −0.03–0.37), and the same was true for SM (*r* = 0.21; *p* < 0.01; bootstrap estimate of the 95% CI: 0.008–0.41). These positive correlations express slightly faster responses after stimulus repetition for higher levels of WM and SM (the dependent variables are inverted latencies for Eriksen and Simon in all models, thus higher scores represent better performance).

### Working memory capacity, secondary memory, and their relationship to fluid intelligence

The relation between WMC and fluid intelligence received considerable attention (Ackerman et al., 2005) and there is consensus that this relation is very strong, though not perfect (i.e., Kyllonen and Christal, 1990; Kane et al., 2005; Oberauer et al., 2005). Unsworth et al. (2009) showed that the relation between complex span and fluid intelligence is mediated in part through SM and in part through PM. Because our indicators of WMC—with the exception of complex span—reflect primarily the capacity of working memory or PM, we understand Unsworth's two-component model as implying that SM should increment the prediction of Gf over and above WMC. The executive attention view and the binding theory both predict that WMC will show unique contributions to the explanation of Gf and both views are agnostic toward a potential increment in the prediction from SM.

**Figure 8:**
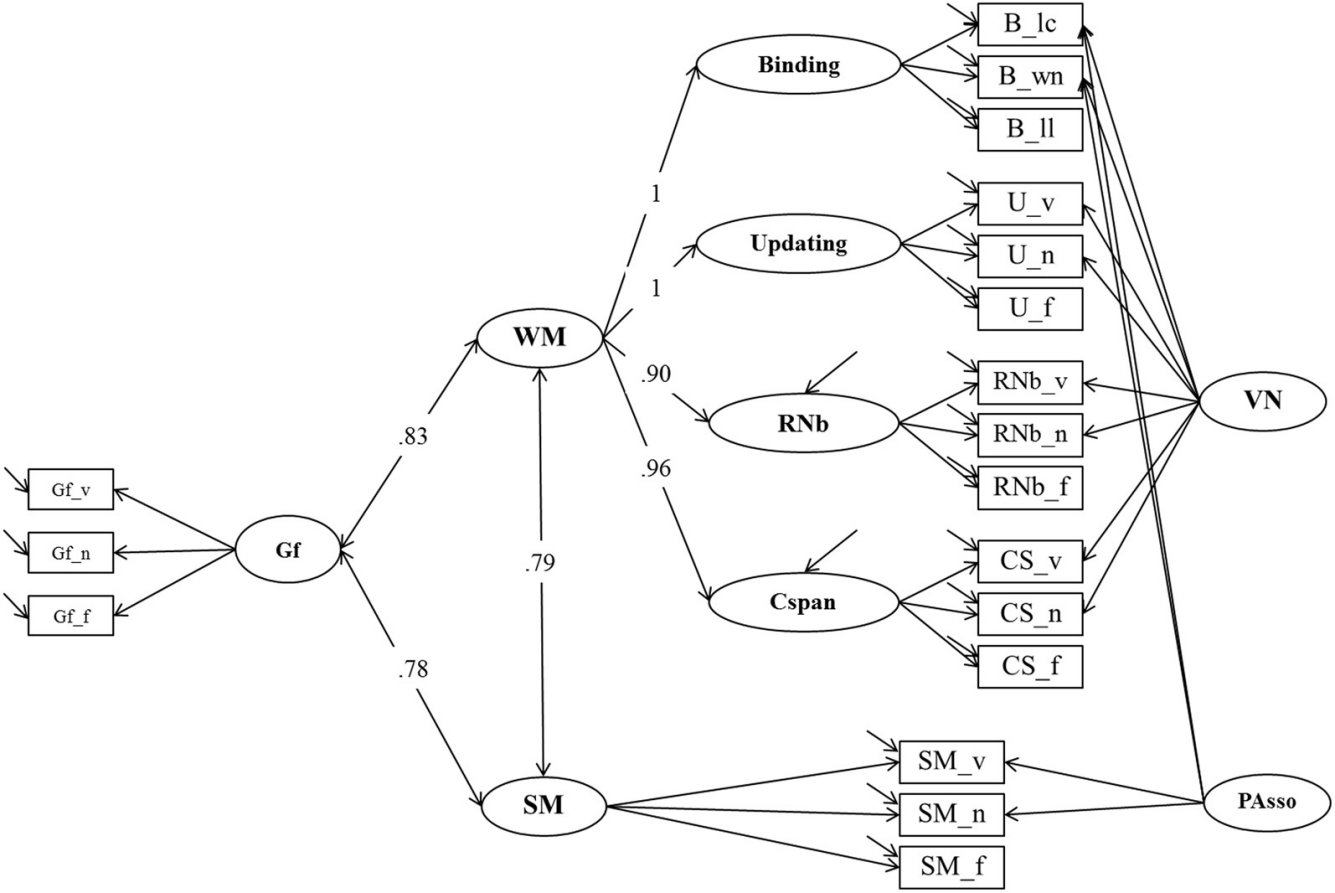
**Structural Equation Model testing the relationship of WM, SM and Gf (Model 5).** Binding, Updating, Recall 1-back (RNb) and Complex Span (Cspan); VN, Verbal-Numerical Content Factor; SM, Secondary Memory; PAsso, Learning Paired-Associations; Gf, Fluid Intelligence; _v, verbal indicators; _n, numerical indicators; _f, figural indicators; _lc, letter-color; _wn, word-number; _ll, location-letter; CS, Cspan indicators.

We extended Model 3 with a further latent factor Gf, measured with three reasoning tests. All correlations between the factors for WMC, SM, and Gf were estimated. The extended model (*Model 5*, depicted in Figure 8) had a good fit: χ^2^[118] = 174.77, *p* < 0.001, CFI = 0.978, RMSEA = 0.043, SRMR = 0.036 (bootstrap *p*-value for the χ^2^-test computed on a single dataset was 0.034; χ^2^[118] = 176.62). Standardized factor loadings of Gf indicators were 0.71 (verbal reasoning), 0.70 (numerical reasoning), and 0.68 (figural reasoning). Loadings of the WMC and SM indicators were highly similar to those estimated in Model 3. WMC and SM were substantially correlated (*r* = 0.79, consistent with Model 3). Both WMC and SM were highly correlated with reasoning: *r* = 0.83 (bootstrap estimate of the 95% CI: 01.77–0.90) and *r* = 0.78 (bootstrap estimate of the 95% CI: 0.67–0.89), respectively. Corresponding with the overlapping confidence intervals, constraining the correlations of WMC with Gf and of SM with Gf to equality did not significantly impair model fit: Δ χ^2^[1] = 0.82; *p* = 0.63. Therefore, the small numerical difference between the freely estimated correlations is—given the statistical power in the present study—inferentially not meaningful.

**Figure 9:**
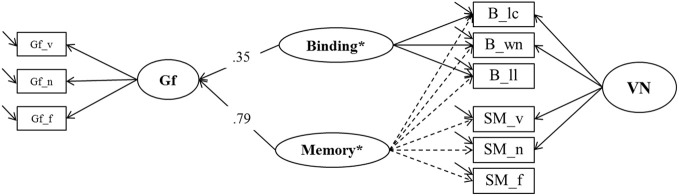
**A first structural model estimating explained variance in Gf by means of binding and secondary memory indicators (Model 6a).** Gf, Fluid Intelligence; VN, Verbal-Numerical Content Factor; _v, verbal indicator; _n, numerical indicator; _f, figural indicator; _lc, letter-color; _wn, word-number; _ll, location-letter.

To test a specific prediction derived from the *binding hypothesis* (Oberauer et al., 2007) we estimated two further models contrasting binding in WM and association memory in SM as predictors of Gf. In the first one (*Model 6a*, depicted in Figure 9), a general Memory factor, which we label Memory^*^, accounted for the common variance of binding and SM indicators. The residual variance common to all indicators of binding (and only those) was accounted for by a specific factor we label Binding^*^ that is orthogonal to Memory^*^. Shared method-specific variance was modeled in a second nested factor (that we label PAsso in line with interpretation from prior models) that was also orthogonal to the other factors in Model 6a. A factor of fluid intelligence (Gf) was regressed on Binding^*^ and Memory^*^. Model fit was excellent: χ^2^[18] = 21.00, *p* = 0.27, CFI = 0.997, RMSEA = 0.025, SRMR = 0.023. The two predictors explained 74% of the variance in Gf. The standardized regression weight of the general Memory^*^ factor was 0.79, and the one originating from the Binding^*^ factor was 0.35. Fixing the regression weight of Binding^*^ to zero impaired model fit significantly: Δ χ^2^[1] = 7.83; *p* < 0.01, showing that the explained Gf variance through residual binding variance over and above the Memory^*^ factor is statistically significant.

**Figure 10:**
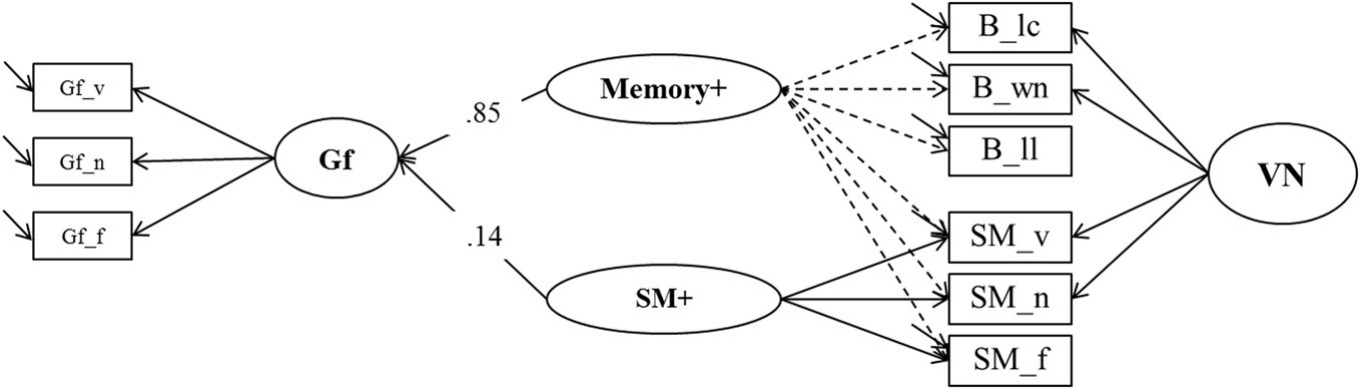
**A second structural model estimating explained variance in Gf by means of binding and secondary memory indicators (Model 6b).** SM+,Secondary Memory for Paired Associations; Gf, Fluid Intelligence; VN, Verbal-Numerical Content Factor; _v, verbal indicator; _n, numerical indicator; _f, figural indicator; _lc, letter-color; _wn, word-number; _ll, location-letter; the residual variance of the SM_n indicator was fixed to zero.

In the second model (*Model 6b*, depicted in Figure 10) we reversed the roles of the binding and the SM indicators. In this model the general factor, which we now label Memory+, was accompanied by a nested factor representing the residuals of the secondary-memory tasks, SM+. In order to achieve convergence in this model the residual variance of the numerical SM indicator was fixed to zero. Model fit was again excellent: χ^2^[19] = 19.53, *p* = 0.42, CFI = 0.999, RMSEA = 0.010, SRMR = 0.023. The explained variance in Gf was 75%, and the standardized regression weight of the general factor Memory+ was 0.85, whereas that of SM+ was 0.14. Fixing the regression weight of SM+ to zero did not impair model fit significantly (Δ χ^2^[1] = 2.54; *p* = 0.12), showing that the specific variance of SM that is not shared with maintenance of temporary bindings did not contribute incrementally to the prediction of fluid intelligence.

## Discussion

Our finding speak to three interrelated questions: What is working memory capacity? How should it best be measured? And finally, how is WMC related to other cognitive constructs, in particular SM, fluid intelligence, and executive attention / cognitive control? We discuss the implications of our findings for these three questions in turn.

### What is working-memory capacity?

#### The case for a general factor

We began by testing to what extent different working-memory task classes reflect the same WMC construct. The hypothesis that complex-span performance reflects to a large extent the ability to retrieve efficiently from SM (Unsworth and Engle, 2007a,b) motivated the prediction that a Complex Span factor is more indicative of SM than the other two paradigm-specific working-memory factors, Recall-N-Back, and Updating, because the latter two paradigms arguably minimize the potential contribution of SM. What we found in Model 1 were strong correlations of performance at the level of latent variables implying a strong overlap and congruence of the constructs measured by the four task classes. Therefore, it is unlikely that the task classes capture SM to very different degrees. Importantly, the present data support the position that Recall-1-back tasks as a specific version of so-called n-back are valid measures of WMC. This result reinforces prior reports (Shelton et al., 2007; Schmiedek et al., 2009) and alleviates the concern raised by Kane et al. (2007) that n-back tasks don't measure the same construct as complex-span tasks. Our findings go beyond prior research by showing very high construct overlap of Recall-N-Back and Complex Span and a nearly perfect relationship with the factor for Updating tasks. Importantly, this conclusion—as the ones to be discussed next—is based on latent variables and not on the analysis of single tasks.

The strong construct overlap between Complex Span and Updating is of particular interest because working-memory updating has been regarded as one of the three factors of executive functions identified by Miyake et al. (2000). The updating factor was highly correlated (0.61) with one complex-span measure (Operation Span) in that study, consistent with our finding. At first glance this close relationship could be interpreted as supporting the executive-attention theory of WMC. We believe that this conclusion would be premature. As pointed out by Ecker et al. (2010), performance in working-memory updating tasks reflects a mixture of variance in general working-memory processes (i.e., keeping available a set of representations over short periods of time, and retrieving them accurately) and variance in the specific efficiency of updating (i.e., substituting old working-memory contents by new ones). When Ecker and colleagues separated these components, they found that only the general working-memory components were related to measures of WMC. Therefore, we interpret the close correlation between the Updating factor and the other WMC factors in our study as reflecting variance in those general working-memory components of updating tasks.

#### Working-memory capacity and binding

Model 2 served to test the binding hypothesis of WMC (Oberauer et al., 2007) by asking how well the general WMC construct—measured through Complex Span, Recall-N-Back, and Updating—is correlated with a Binding factor. In this model the general WMC factor was perfectly correlated with the Binding factor, so that the loading of Binding on WMC could be fixed to 1 without loss of fit. The WMC factor also accounted for 100% of the Updating variance, 79% of the Recall-1-back variance, and 90% of the variance in Complex Span. These findings suggest that the common source of variance across all four task paradigms is the cognitive mechanism of building, maintaining and updating arbitrary bindings (Oberauer, 2009; Oberauer et al., 2007).

Rapid formation and updating of bindings is needed for the Updating and Recall-1-back tasks because accurate performance in these paradigms requires memory for the relations between items and their contexts (e.g., relations between letters and locations, or between words and categories), and rapid updating of those relations. Bindings are also a core factor for success in Complex Span tasks, because these tasks require the recall of items in the correct serial order (Schmiedek et al., 2009; Oberauer et al., 2012). For the recall of serial positions in a list it is necessary to create firm bindings between content (words, letters, length and directions of arrows) and context (the serial position of a word, letter, or arrow within the list). Additionally, these bindings need to be established and maintained in the presence of an interfering secondary task.

### How to measure working memory capacity

#### Psychometric considerations

One corollary of the strong correlation between different working-memory paradigms is that all four task classes can be seen as good proxies of a general WMC factor. A closer look at the psychometric quality and attributes of competing paradigms allows for a more refined perspective.

First, concerning the magnitude of the loadings of the task-class specific factors on the second order WMC factor, the Binding and Updating factors did best, showing no task-specific variance at all, and Complex Span did comparatively less well, showing the largest amount of task-class specific variance. These differences notwithstanding, all four task classes are good indicators of WMC, because they reflect to a large extent reliable variance of the general WMC construct. The relatively novel Recall-N-back paradigm is arguably a very efficient method for assessing WMC because it enables continuous recording of performance at a high rate.

Second, the relevance of content factors in this broad task battery seems to be weaker than has been assumed by some authors (Shah and Miyake, 1996; Süßet et al., 2002). It is possible that the relevance of content variance is larger for conventional short term memory span measures, such as digit span or letter span, than for working-memory tasks (Kane et al., 2004).

Third, the binding tasks had shared variance with the SM task, which could be taken to compromise the clean separation of measurement of WMC and SM. The covariance between our binding tasks and the paired-associates learning tasks that we used as indicators of SM probably reflect the shared method variance between these two task paradigms. The largely analogous methods for these two paradigms was intended to enable a direct comparison of the ability to maintain temporary bindings in working memory and the ability to acquire more long-term associations in SM (as in our Models 6a and 6b). SM could be measured in a more method-independent way through multiple indicators using different methods.

#### Practical recommendations

Every cognitive test carries some task-specific variance that is unrelated to the construct of interest, and tests of WMC are no exception. Therefore, we generally recommend measuring WMC through a heterogeneous set of paradigms to avoid mono-operation bias (Shadish et al., 2002; Lewandowsky et al., 2010). Often, however, only limited time and resources are available to measure WMC, such that administration of only a single test is feasible. In light of our findings of high correlations between four different WMC paradigms, this is a defensible practice. In that case, a number of considerations can be made to choose among the available paradigms.

First, the four task classes investigated here differ in their construct validity, as reflected in their loadings on the general WMC factor. Although the differences were not large, they might weigh slightly in favor of using the Binding or the Updating task rather than Cspan or Recall-N-Back. Second, the tasks differ in their efficiency, that is, the number of independent measurements per testing time. Complex-span trials take relatively long, whereas trials of the other three paradigms are shorter, which means that a more reliable score can be obtained in the same amount of time (see Table A2 for details). A third consideration concerns the exhaustiveness and sufficiency of task scores. In all complex-span tasks participants work on two tasks, a memory task and a concurrent processing task, but only their memory performance is considered in scoring. Although cut-off scores in processing-task performance are usually applied (Conway et al., 2005), odds are that there remain stable individual differences in processing performance which are ignored in scoring the tasks (and also in subsequent data analysis). This issue might be a nuisance in scientific use but is a serious problem in using Complex Span measures as a diagnostic tool in high-stakes contexts. There are also multiple ways of calculating the recall scores (see the procedures discussed in Conway et al., 2005). In contrast, there is less ambiguity of scoring for the other three task classes.

### Working memory capacity in relation to other cognitive constructs

#### Working memory capacity, secondary memory, and fluid intelligence

The results from Model 3 are consistent with previously reported correlations between WMC and SM, showing that they are separable but closely correlated constructs (Unsworth et al., 2009). The correlation reported here is stronger than that reported in previous studies. This might be the case because our WMC factor reflects the common variance of four different working-memory paradigms, whereas it was restricted to Complex Span indicators for working memory in previous work. We conclude that the close relation between WMC and SM is not specific to complex-span tasks, but rather extends to Updating, Recall-N-Back, and Binding tasks. At first glance this finding is surprising because, whereas the complex-span paradigm bears close similarity to established SM tasks such as the continuous-distractor task, the latter three paradigms were designed to minimize the potential contribution of SM: The Updating, Recall-N-Back, and Binding tasks used comparatively short retention intervals, thereby leaving little chance for encoding into SM, and they generated a high level of proactive interference, thereby minimizing the usefulness of SM representations (for a similar argument regarding proactive interference see Cowan et al., 2012). Therefore, it appears implausible that variance in the efficient use of SM plays a major role in determining performance in those working-memory paradigms. Our finding becomes less surprising when we consider the reverse direction of causality: According to the binding hypothesis, high WMC reflects the ability to establish robust bindings in working memory, which in turn support encoding of those bindings into SM. Therefore, high WMC might be a cause, not a consequence, of a well-functioning SM.

At the same time, there is a substantial proportion of unique variance in both SM and the second order WMC factor. Therefore, the present results clearly show that WMC is not equivalent with SM. This conclusion is reinforced by the finding that Binding provides an independent contribution besides SM to predicting fluid intelligence (Models 6a and 6b). One possible interpretation of this finding is that fluid intelligence reflects on the one hand the ability to maintain and update temporary bindings in working memory (Oberauer at al., 2007), and on the other hand the ability to acquire more lasting associations in SM (Tamez et al., 2008, 2012; Kaufman et al., 2009). We tested the relative contribution of bindings in working memory and association-learning in SM to predicting fluid intelligence in two models focusing on Binding and SM as predictors of Gf. Despite the strong collinearity between SM and the Binding factor it seems as if the Binding factor was slightly more important for the prediction of fluid intelligence: Once Binding performance was statistically controlled for, SM was no longer significantly related to fluid intelligence. Although the present results require replication we conclude that the results for Model 3 and the models derived from it are in line with the binding hypothesis of WMC (Oberauer et al., 2007).

#### Working-memory capacity and cognitive control

In Models 4a through 4c we tested the relation between the efficiency of cognitive control and the other factors. The two factors representing the efficiency of cognitive control—conflict costs in the Simon task and in the Eriksen flanker task—were unrelated with each other. This is a replication of previous findings showing that conflict related slow-down factors don't generalize across these two paradigms (Keye et al., 2009, 2010). This result is not due to a lack of reliability of these factors (see Table A2). If both paradigms capture similar conflict costs they should share a substantial amount of variance, and hence should be correlated at least moderately positive with each other, contrary to our finding. We conclude that either at least one of the two conflict cost factors is not a valid measure of cognitive control in response-conflict situations, or that individual differences in cognitive control in response-conflict tasks are entirely task specific. From the perspective of the theory of Executive Attention this results is discouraging because it suggests that measures taken to reflect the ability to cope with interference and distraction essentially capture task-specific variance in performance. We also observed no correlation between the conflict-related slow down factors in either the Simon or the Eriksen task and any of the WM and SM factors. These findings replicate and extend previous reports on the correlation between WMC and factors of conflict costs after removing variance due to individual differences in overall speed (Keye et al., 2009, 2010). Other research on this issue is somewhat inconclusive: Redick and Engle (2006) and Heitz and Engle (2007) found that high WM span participants were faster at minimizing the distracting impact of incompatible flankers as compared to low WM span participants—a result that was not found for the compatible trials (Heitz and Engle, 2007). These results arose from extreme-group comparisons, which are regarded as problematic in individual-differences research (see Preacher et al., 2005). Other studies that did not rely on extreme-group comparison reported no correlation between WMC and performance on the flanker task (Friedman and Miyake, 2004).

The present methodological approach relies on latent variable modeling of factors reflecting cognitive control, partialling out baseline reaction time along with other potentially confounded response components such as repetition priming. In addition, we used a broad battery of working memory measures to measure WMC on a high level of generality. We found that any relation of both the Simon and the Eriksen paradigm with WMC is entirely due to individual differences in overall choice reaction time. This relation is probably best understood as an instance of the correlation between WMC and general cognitive speed in choice tasks (Schmiedek et al., 2007).

## Conclusions

To conclude, we obtained strong evidence for the binding hypothesis of WMC: This hypothesis correctly predicted that complex-span tasks, updating tasks, and binding tasks all shared a large proportion of variance, which reflects a broad general WMC construct and strongly predicts fluid intelligence. We also obtained some evidence for the two-component hypothesis of Unsworth and Engle (2007a,b). This hypothesis correctly predicted that a measure of SM is closely related to complex span and to fluid intelligence. This hypothesis did not predict, but is at least compatible with the finding that SM was equally strongly correlated with our updating and binding tests. Finally, our results are not well explained by the executive-attention theory of WMC, which erroneously predicts a close correlation between measures of inhibition one the one hand, measures of WMC and fluid intelligence on the other hand.

## Author notes

Correspondence and requests for reprints should be addressed to Oliver Wilhelm, Department of Psychology, University Ulm, e-mail: oliver.wilhelm@uni-ulm.de We thank Kriszta Groß, Astrid Kiy, Karsten Manske, Janina Künecke, Carolyn Nelles, Stephanie Mayer and Alf Mante for help with data acquisition. This research was supported by a grant from the Deutsche Forschungsgemeinschaft (Wi 2667/4-2) to Oliver Wilhelm and Klaus Oberauer.

### Conflict of interest statement

The authors declare that the research was conducted in the absence of any commercial or financial relationships that could be construed as a potential conflict of interest.

## Footnotes

^1^One limitation of this study was that reliability estimates, inter-task correlations, and factor loadings for several of the measures were low. Miyake and Friedman (2012) recently provided an update on their model, which has better psychometric properties.

^2^One study separated these two sources of variance (Ecker et al., 2010): these authors found that working memory updating tasks correlated with a WMC factor (measured through non-updating tasks) primarily because they involved short-term maintenance and processing, whereas the updating process itself (referred to as substitution) did not correlate with WMC.

^3^It is common to control for effects of task order (including effects of item order and block order) in experimental settings but very uncommon to do so when the purpose of testing is to measure person parameters (see Wilhelm and Oberauer, 2006 for a regression analytic application with binding measures). Person parameters are compromised if there are individual differences in response to task order variations. Depending on the nature of task-order effects (effects of position, of order, and carry-over effects ought to be distinguished), their magnitude, and their relevance Yousfi and Böhme (2012) recommend sophisticated and flexible item response theoretic solutions, but these solutions require many more test items than were available in the present study. Therefore we cannot rule out effects of the specific task order. Any such effects, however, should inflate correlation estimates in the same way for all correlations, without distorting the pattern of correlations that is critical for our structural analyses.

^4^Due to moderate floor and ceiling effects in some of the tasks (see Table A2 for details) we re-estimated all models based on datasets for which data points at floor (<0.08) and ceiling (>0.95), which caused the skewness and non-normality of those distributions, were replaced by imputed data points based on an imputation model that included all variables (see Polasek and Krause, 1994). The multiple imputations of datasets were realized with the *R* package mice by van Buuren and Groothuis-Oudshoorn (2011) using a routine implemented by Robitzsch (2013). Estimating all models with these imputed data had only small effects on correlation and regression coefficients and had no impact on the conclusions drawn from the models.

## Appendix

**Table A1:** **Classification of the indicators**.

**Name of indicator**	**Serial position**	***N* items load a**	***N* items load b**	***N* items load c**	***N* items load d**	***N* items load e**	***N* items all**
Binding verbal	02/S1	2 (2)	3 (3)	3 (4)	3 (5)	4 (6)	15
Binding verbal-num.	04/S2	2 (2)	4 (3)	4 (4)	3 (5)	1 (6)	14
Binding figural	09/S2	1 (2)	4 (3)	4 (4)	3 (5)	2 (6)	14
Updating verbal	13/S2	2 (2)	3 (3)	4 (4)	3 (5)	–	12
Updating numerical	04/S1	2 (2)	2 (3)	3 (4)	3 (5)	2 (6)	12
Updating figural	02/S2	3 (2)	3 (3)	3 (4)	2 (5)	–	11
Recall 1-back verbal	15/S2	2 (1)	5 (2)	5 (3)	–	–	12
Recall 1-back num.	05/S2	2 (1)	5 (2)	5 (3)	–	–	12
Recall 1-back figural	07/S1	2 (1)	5 (2)	5 (3)	–	–	12
Reading span	03/S2	3 (2)	3 (3)	3 (4)	3 (5)	–	12
Operation span	08/S2	3 (2)	3 (3)	3 (4)	3 (5)	–	12
Rotation span	05/S1	3 (2)	3 (3)	3 (4)	3 (5)	–	12
LTM verbal	06/S1 12/S2	2 (20)	–	–	–	–	2
LTM verbal-num.	08/S1 07/S2	2 (20)	–	–	–	–	2
LTM figural	01/S2 16/S2	2 (12)	–	–	–	–	2
Reasoning verbal	10/S1	–	–	–	–	–	16
Reasoning num.	10/S1	–	–	–	–	–	16
Reasoning figural	10/S1	–	–	–	–	–	16
Simon	06/S1 08/S1	–	–	–	–	–	2 × 80
Eriksen	07/S2 12/S2	–	–	–	–	–	2 × 80

Note. Load-levels are displayed in brackets.

**Table A2:** **Descriptive statistics—all performance indicators**.

**Name of indicator**	***M***	***SD***	α	ω
Binding verbal	0.64	0.12	0.69	0.70
Binding verbal-numerical	0.68	0.14	0.79	0.80
Binding figural	0.81	0.13	0.82	0.82
Updating verbal	0.66	0.16	0.81	0.82
Updating numerical	0.73	0.17	0.85	0.85
Updating figural	0.74	0.15	0.72	0.72
Recall 1-back verbal	0.72	0.20	0.94	0.94
Recall 1-back numerical	0.63	0.20	0.94	0.94
Recall 1-back figural	0.44	0.16	0.93	0.93
Reading span	0.79	0.19	0.89	0.89
Operation span	0.89	0.13	0.88	0.88
Rotation span	0.68	0.18	0.83	0.84
Secondary Memory verbal	0.41	0.26	0.94	0.94
Secondary Memory verbal-numerical	0.24	0.16	0.85	0.85
Secondary Memory figural	0.25	0.16	0.74	0.74
Reasoning verbal	0.53	0.16	0.62	0.62
Reasoning numerical	0.51	0.17	0.63	0.63
Reasoning figural	0.43	0.18	0.67	0.68
Simon _cC_id^*^	2.17	0.30	0.91	0.91
Simon _cC_ni^*^	1.96	0.34	0.91	0.91
Simon _iC_id^*^	1.93	0.31	0.92	0.91
Simon _iC_ni^*^	1.81	0.33	0.92	0.92
Simon _cI_id^*^	1.73	0.32	0.93	0.93
Simon _cI_ni^*^	1.66	0.32	0.92	0.92
Simon _iI_id^*^	1.91	0.29	0.91	0.91
Simon _iI_ni^*^	1.75	0.32	0.93	0.93
Eriksen _cC_id^*^	2.08	0.26	0.90	0.90
Eriksen _cC_ni^*^	2.02	0.29	0.95	0.95
Eriksen _iC_id^*^	2.00	0.27	0.94	0.94
Eriksen _iC_ni^*^	1.98	0.26	0.93	0.93
Eriksen _cI_id^*^	1.68	0.21	0.93	0.94
Eriksen _cI_ni^*^	1.73	0.28	0.95	0.95
Eriksen _iI_id^*^	1.74	0.23	0.92	0.92
Eriksen _iI_ni^*^	1.75	0.24	0.93	0.94

*Note*.

* M, SD and Reliability was calculated for inverted latencies; cC, compatible trials following compatible trials; iC, compatible trials preceded by incompatible trials; cI, incompatible trials following compatible trials; iI, incompatible trials preceded by incompatible trials; _id, identical (Repetition Priming); _ni, non-identical (Unprimed); α and ω have been calculated with the R package psych Revelle (2013).
